# Continuous polarization–wavelength mapping with nonlocal metasurfaces

**DOI:** 10.1038/s41377-026-02233-5

**Published:** 2026-03-13

**Authors:** Jiuxu Wang, Jie Wang, Feilong Yu, Jin Chen, Rongsheng Chen, Tianxiong Geng, Rong Jin, Yiran Zhou, Tongwen Zheng, Guanhai Li, Xiaoshuang Chen, Wei Lu

**Affiliations:** 1 State Key Laboratory of Infrared Physics, Shanghai Institute of Technical Physics, Chinese Academy of Sciences, Shanghai, China; 2 University of Chinese Academy of Sciences, Beijing, China; 3 Hangzhou Institute for Advanced Study, University of Chinese Academy of Sciences, Hangzhou, China; 4 Shanghai Research Center for Quantum Sciences, Shanghai, China

**Keywords:** Nanophotonics and plasmonics, Metamaterials

## Abstract

Simultaneous and continuous control over polarization and wavelength—two orthogonal and information-rich degrees of freedom—remains a central challenge in metasurface photonics, long hindered by intrinsic dispersion constraints and structural degeneracy. Here, we customize continuous polarization–wavelength mapping through a nonlocal metasurface platform that decouples birefringent evolution from structural dispersion. We achieve programmable, spectrally resolved polarization shaping across the broadband mid-infrared regime by introducing an equivalent nonlocal Jones matrix formalism and a dimension-interlaced vectorial diffraction neural network. This framework enables fully continuous and arbitrarily prescribed mapping across the joint polarization–wavelength space—beyond the capabilities of segmented or interleaved metasurface designs. We experimentally demonstrate non-degenerate multicolor vectorial holography, broadband achromatic imaging, and arbitrary elliptical polarization multiplexing with high fidelity and minimal crosstalk, maintaining strong channel isolation. Our results establish a scalable route toward continuous-domain photonic encoding, offering a powerful foundation for ultracompact optical communication, vectorial information encryption, and high-dimensional light-field processing.

## Abstract (web summary)

Continuously spectral-encoded polarization shaping enabled by nonlocal metasurfaces

## Graphical abstract

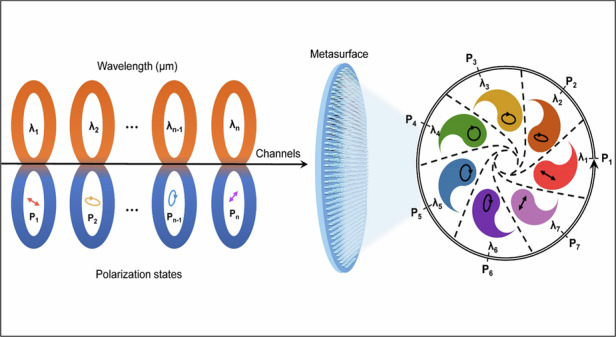

## Introduction

Encoding and manipulating optical information across multiple physical dimensions is central to emerging demands in quantum-secure communication, in-sensor computing, and ultra-dense photonic interconnects. Among the various degrees of freedom (DoFs) of light, polarization and wavelength are particularly attractive: they are intrinsically orthogonal, continuous, and highly robust, making them ideal carriers for high-dimensional photonic encoding. Their combined space defines an infinite-dimensional Hilbert basis, perfectly suited to support tasks ranging from quantum-secure key distribution to in-sensor neural inference and spatiotemporal signal reconstruction^1–6^. Jointly accessing and controlling these DoFs, however, remains a formidable challenge. Existing strategies typically treat polarization and wavelength independently, using cascaded components, segmented zones, or multilayer assemblies. Such fragmented architectures introduce loss, complexity, and crosstalk, and fundamentally restrict modulation to discrete, pre-defined states^7–11^.

In contrast to traditional optical systems that manipulate photonic DoFs through segmented architectures—splitting functions across different spatial regions, time slots, or cascaded components—metasurfaces offer a fundamentally different paradigm: simultaneous, co-located, and loss-minimized control of multiple DoFs on a single planar interface^12–17^. This physical integration has catalyzed a wave of breakthroughs in multifunctional wavefront shaping, including multi-foci metalenses^18^, field-of-view tunable meta-systems^19,20^, varifocal meta-devices for augmented-reality displays^21^, quantitative phase imaging for meta-lenses and large-depth-of-field imaging^22,23^ and vectorial holography^24–29^. By engineering meta-atoms with tailored phase and birefringent responses, these works have demonstrated compact optical systems with unprecedented functionality^30–33^. Moreover, recent advances in active and hybrid metasurfaces have enabled quasi-continuous spectral control through phase-change materials, carrier doping, electro-optic modulation, and multi-resonant or BIC-assisted coupling mechanisms^34–40^. These developments substantially expand the design freedom of dispersion engineering. However, such approaches typically rely on external stimuli or discrete resonant states, whereas there have been no reports on intrinsic, passive, and physically continuous modulation across the polarization-wavelength domain. Recent advances in wavelength-selective polarization digital metasurfaces have demonstrated multiplexed encoding of polarization-dependent holographic channels across discrete spectral bands, offering an insightful exploratory effort to approach continuous-domain multidimensional photon control^41^.

Additionally, current metasurface designs remain fundamentally constrained to discrete or quantized modes of control. This stems from two intertwined physical bottlenecks: (1) the quasi-linear dispersion curvature inherent to structural resonances, which restricts the degrees of freedom available for spectral control; and (2) the degeneracy of the Jones matrix in symmetric or weakly anisotropic structures, which binds the polarization and wavelength channels into inseparable combinations. As a result, prior efforts—no matter how sophisticated such as spatial interleaving of unit cells, composite high-aspect-ratio meta-atoms, or stacked diffractive layers—operate within a fixed discrete grid of polarization and wavelength states, fundamentally precluding continuous, independent, and reconfigurable access to the full parameter space^42–49^.

In this work, we present a nonlocal metasurface platform that breaks this long-standing bottleneck and enables physically continuous polarization–wavelength mapping. By introducing an equivalent nonlocal Jones matrix formalism and a forward model linking structural perturbations to far-field vectorial responses, we decouple polarization evolution from spectral dispersion. A dimension-interlaced vectorial diffraction neural network compresses the high-dimensional design space from *4n*^*3*^ to *8n*, enabling efficient joint optimization of rotation angle and wavelength across continuous-domains. This framework allows for programmable, high-fidelity manipulation of arbitrary polarization–wavelength mappings, beyond the capabilities of segmented or interleaved metasurfaces.

We experimentally validate this platform with multicolor vectorial holography, continuous gradient-polarization imaging, and arbitrary elliptical polarization multiplexing in the mid-infrared. The demonstrated fidelity, crosstalk suppression, and broadband robustness confirm the viability of our approach. Table 1 shows the comparison of different metasurface design in realizing the high-dimensional multiplexing. It implies that our work marks a meaningful step in metasurface photonics—from discrete channel engineering to continuous-domain, multidimensional light-field control—offering a scalable foundation for future optical systems in communication, encryption, and intelligent sensing.

**Table 1:** Comparison of different metasurfaces in high-dimensional multiplexing

Design method	Polarization channels	Wavelength channels	Arbitrary channels	I/O form	Refs
Multifocal plane	2 2	3 10	Nonarbitrary	Fixed input, variable output	^41,42,46^
Interleaved	1	3	Nonarbitrary	Fixed input, variable output	^49^
Pixelated	4 1	3 Continuous	Arbitrary Arbitrary	Fixed input, variable output	^47,48^
Multilayer	9	2	Nonarbitrary	Fixed input, variable output	^44^
Complex units	6	3	Nonarbitrary	Fixed input, variable output	^29^
Neural networks	9	3	Nonarbitrary	Fixed input, variable output	^28^
Continuous Reconstruction	Continuous	Continuous	Arbitrary	Variable input, variable output	This work

## Results

**Fig. 1:**
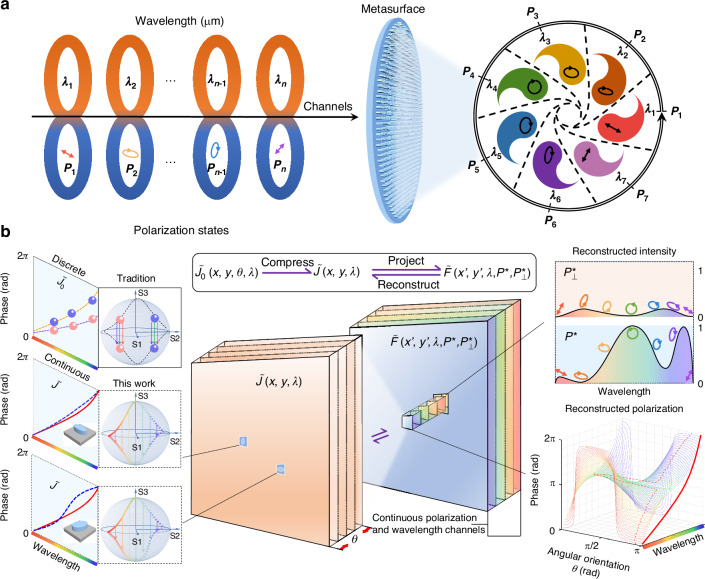
Conceptual framework and modeling pipeline of continuous information domain reconstruction. **a** Schematic illustration of a nonlocal metasurface enabling continuous-polarization–wavelength holography. Each input polarization state, corresponding to a specific wavelength, is mapped via a continuous polarization channels on the Poincaré sphere across the metasurface, producing a broadband gradient polarization hologram. **b** The proposed dimension-interlaced continuous design framework. Due to the nearly constant group delay of local resonant modes (yellow/purple dashed lines), conventional metasurfaces are limited to quasi-linear dispersion, leading to discrete and degenerate Jones responses. By introducing a nonlocal equivalent Jones matrix and perturbation-driven dispersion modeling, we enable the far-field reconstruction of arbitrary polarization–wavelength responses. The left side shows the physical limitation of local metaatoms under first-order dispersion. The right side illustrates how the reconstructed nonlocal response (blue dashed line) achieves precise field matching across continuous spectral and polarization trajectories

To overcome the inherent limitations in controlling photonic degrees of freedom—particularly the degeneracy between polarization and wavelength imposed by local dispersion—we propose a dimension-interlaced continuous design framework as illustrated in Fig. 1. As shown in Fig. 1a, we construct a nonlocal metasurface capable of generating continuous gradient polarization holography across the mid-infrared spectrum (2.7–4 μm), with each polarization state smoothly evolving with wavelength. Unlike conventional multiplexing strategies that discretely combine polarization–wavelength pairs, this design realizes continuous-domain modulation by reconstructing the far-field response from a nonlocal equivalent Jones matrix.

In Fig. 1b, we illustrate how conventional metasurface designs are constrained by the nearly constant group delay of structural eigenmodes (depicted by yellow and purple dashed lines on the left insert), which results in quasi-linear phase dispersion and limits the curvature (${\partial }^{2}\varphi /\partial {\lambda }^{2}$) needed for broadband, arbitrary modulation. The dispersion Jones matrix of such local structures can be expressed as:

1 $${\widetilde{J}}_{0}\,\left(x,\,y,\theta ,\lambda \right)=R\left(-\theta \right){\rm{\cdot }}\left(\begin{array}{cc}{t}_{\alpha }\left(\lambda \right)\cdot {e}^{i{\varphi }_{\alpha }\left(\lambda \right)} & 0\\ 0 & {t}_{\beta }\left(\lambda \right)\cdot {e}^{i{\varphi }_{\beta }\left(\lambda \right)}\end{array}\right)R\left(\theta \right)$$

Here, θ is the rotation angle of the meta-atom, and t_α,β_, φ_α,β_ represent the amplitude and phase responses of the two eigen-polarization channels. Under the traditional C2-symmetrical structure with linear phase dispersion, θ becomes wavelength-invariant, precluding complex polarization evolution across spectrum.

To overcome this, we define a nonlocal equivalent Jones matrix by incorporating the spatial diffraction kernel f(x′, y′, z, λ), yielding:

2 $${\widetilde{J}}^{{\prime} }\left({x}^{{\prime} },{y}^{{\prime} },\theta \left(\lambda \right),\lambda \right)={\iint }_{x,y}\,{\widetilde{J}}_{0}\,\left(x,\,y,\theta ,\lambda \right)\cdot f\left({x}^{{\prime} }-x,{y}^{{\prime} }-y,z,\lambda \right){dxdy}$$

This formalism introduces spatial-frequency dispersion and enables phase modulation beyond the local approximation, effectively unlocking an additional DoF associated with eigenvector evolution across the polarization–wavelength manifold. To link these responses to designable structural parameters, we construct a forward analytical model that relates the birefringent phase retardation *Δφ* = *φ*_*β*_ − *φ*_*α*_ to the target polarization path defined by ellipticity P_1_(λ) and azimuth P_2_(λ):

3 $$tan\left(-\frac{\Delta \varphi (\lambda )}{2}\right)=\frac{tan\left(2{P}_{1}\left(\lambda \right)\right)}{sin2({P}_{2}(\lambda )+\theta \left(\lambda \right))}$$

This expression allows us to extract the optimal rotation angle θ(λ) needed to reproduce any desired polarization trajectory on the Poincaré sphere (see Supplementary Note 3 and 4 for details). Using this perturbative mapping, we then define the far-field projection intensity along the conjugate polarization state as:

4 $$E\left({x}^{{\prime} },{y}^{{\prime} },\lambda \right)={\iint }_{x,y}\,\left\langle \left.{P\left(\lambda \right)}^{* }\right|\cdot {\widetilde{J}}_{0}\,\left(x,\,y,\theta ,\lambda \right)\,\cdot \left|P\left(\lambda \right)\right.\right\rangle \cdot f\left({x}^{{\prime} }-x,{y}^{{\prime} }-y,z,\lambda \right){dxdy}$$

5 $${E}_{\perp }\left({x}^{{\prime} },{y}^{{\prime} },\lambda \right)={\iint }_{x,y}\,\left\langle \left.{{P}_{\perp }\left(\lambda \right)}^{* }\right|\cdot {\widetilde{J}}_{0}\,\left(x,\,y,\theta ,\lambda \right)\,\cdot \left|P\left(\lambda \right)\right.\right\rangle \cdot f\left({x}^{{\prime} }-x,{y}^{{\prime} }-y,z,\lambda \right){dxdy}$$

These fields form the basis of the nonlocal Jones response in the focal plane. To represent the dispersion behavior of the metaatoms, we further introduce a third-order polynomial model for both amplitude and phase:

6 $${E}_{m}\left(n,\theta ,\lambda \right)=\left[a\cdot {\left(1/\lambda \right)}^{3}+b\cdot {\left(1/\lambda \right)}^{2}+c\cdot 1/\lambda +d\right]\cdot {e}^{i\cdot \left[{ap}\cdot {\left(1/\lambda \right)}^{3}+{bp}\cdot {\left(1/\lambda \right)}^{2}+{cp}\cdot 1/\lambda +{dp}\right]}$$

Each meta-atom is characterized by a discrete set of coefficients [a, b, c, d, ap, bp, cp, dp]. By combining this model with the polarization path projection, we construct two hierarchical optimization layers—one for polarization channel matching and one for phase-dispersion perturbation fitting. This reduces the data complexity from 4n^3^ to 8n, enabling fast and globally optimal inverse design across a continuous polarization–wavelength parameter space (see Supplementary Note 5 for details). Together, this framework enables the first metasurface design strategy capable of simultaneously and densely sampled continuous controlling of polarization and wavelength, marking a transition from discrete channel manipulation to continuous-domain optical field engineering. Notably, the continuity here does not refer to an infinite number of experimentally resolvable states, but rather to a mathematically continuous mapping between input and output polarization–wavelength states defined by the nonlocal Jones-matrix formalism (Eq. 4). The Supplementary Note 2 provides more details.

**Fig. 2:**
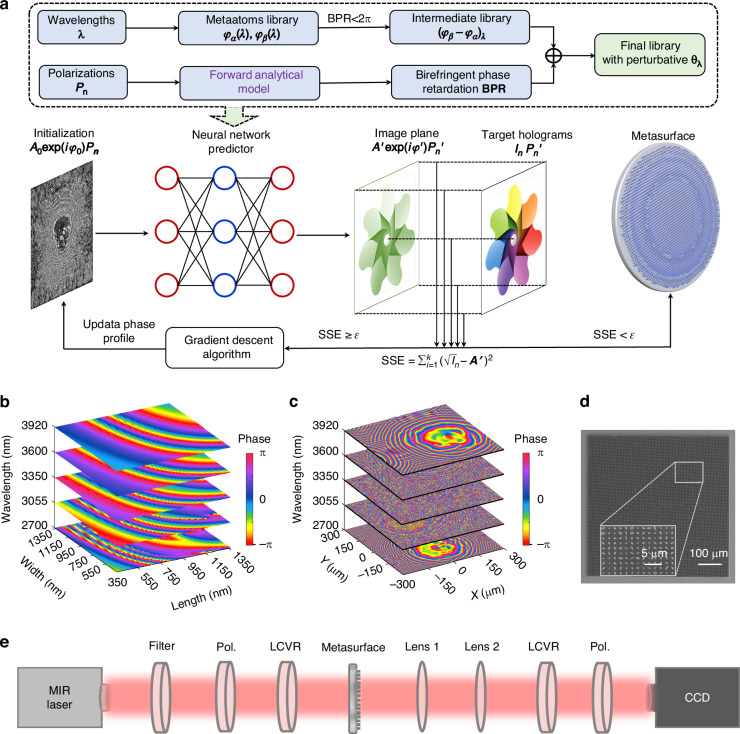
Implementation pipeline of the dimension-interlaced continuous design framework. **a** Schematic flowchart of the proposed metasurface inverse-design strategy, combining a continuous polarization–wavelength analytical model with a dimension-interlaced vectorial diffraction neural network. **b** Simulated phase library of silicon meta-atoms at five representative wavelengths. The unit cell has a period of 1.5 μm and a height of 7 μm. **c** Optimized multi-wavelength phase profiles under different weighting factors, accounting for broadband loss compensation. **d** SEM image of the fabricated metadevice (lateral size: 600 μm), demonstrating high-aspect-ratio structures. **e** Optical setup for broadband polarization-resolved holographic characterization. LCVR: liquid crystal variable retarder and polarizer for polarization generation and analysis

The complete design and implementation flow of our dimension-interlaced continuous design framework is illustrated in Fig. 2. As shown in Fig. 2a, we integrate a continuous polarization–wavelength analytical model with a dimension-interlaced vectorial diffraction neural network, enabling inverse design of nonlocal metasurfaces over high-dimensional parameter spaces. Compared to conventional diffraction neural networks that rely on discrete pixel-wise phase targets, our approach introduces continuous variables in both wavelength and polarization domains, which allows the network to learn the projection relationship between conjugate polarization channels and nonlocal structural responses.

A key methodological innovation lies in the pre-compression of the meta-atom database and the transformation of the design problem into the conjugate polarization basis. Specifically, the effective “feature angle” of each meta-atom is extracted based on its birefringent response, and the projection intensity onto the conjugate polarization channel is computed. This compressed and polarization-aligned representation is then used as the input for the neural network, while the target Jones matrix in the focal plane—also expressed in the conjugate basis—serves as the excitation function. Additionally, a rotation angle perturbation layer is introduced to reflect the polarization-path-dependent phase response, enabling joint optimization over continuous θ and λ dimensions. This formulation not only reduces the optimization parameter space from cubic to quadratic complexity but also facilitates global convergence across the metasurface. The compressed dual-layer neural network architecture captures both the local phase-dispersion matching and global polarization trajectory customization. As a result, the design process becomes highly efficient, scalable, and physically interpretable.

To validate the platform’s physical feasibility, we construct a silicon-based meta-atom library using numerical simulations. As shown in Fig. 2b, the library is built over five representative wavelengths between 2.7–4 μm, covering structures with a fixed period of 1.5 μm and a height of 7 μm. Silicon (*n* ≈ 3.43) is chosen as the structural material due to its low loss in the mid-infrared and strong birefringent potential. With a mature deep reactive ion etching process, we achieve aspect ratios exceeding 20, which is essential for broadband dispersion engineering. Figure 2c presents the optimized phase profiles over the five spectral channels. These phase targets are weighted according to the laser energy attenuation at each wavelength to ensure uniform holographic fidelity across the band. Compared with the intrinsic quasi-linear behavior of raw meta-atom dispersion, the optimized phase profiles exhibit enhanced wavelength nonlinearity after projection into the conjugate polarization space, validating the efficiency of our compression strategy. The fabricated metadevice is shown in Fig. 2d. The 600 μm-sized sample contains over 160,000 anisotropic meta-units patterned with high fidelity. Tolerance analysis shows that deviations in sidewall angle ( < 0.5°), etch depth ( ± 200 nm), and linewidth ( ± 20 nm) introduce minimal influence on birefringent dispersion and overall holographic performance. These findings confirm that the proposed metasurface design is well within the capability of current nanofabrication technologies. The detailed tolerance analysis of fabrication errors can be found in Supplementary Note 6. Figure 2e depicts the optical measurement setup used for verifying holographic and polarization performance. A broadband mid-IR supercontinuum source is collimated, filtered via bandpass elements, and polarization-modulated using a liquid crystal variable retarder and wire-grid polarizers. The holographic output is then captured by an MWIR camera, enabling spatial and polarization-resolved imaging.

**Fig. 3:**
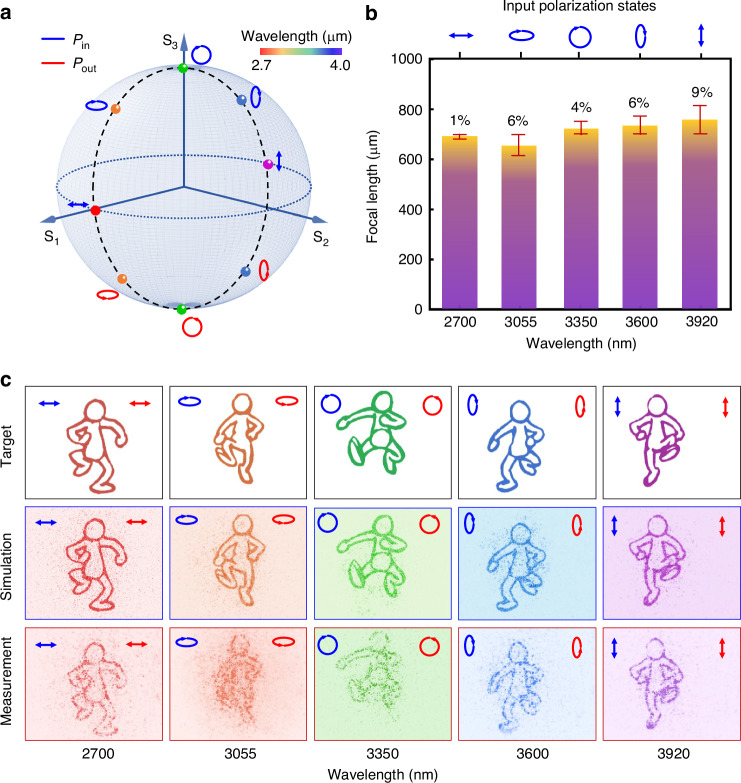
Achromatic characterization of multicolor continuous-domain polarization holography metasurface. **a** Polarization–wavelength mapping on the Poincaré sphere. Five elliptical polarization states (color-coded by wavelength) evolve quasi-continuously along a conjugated path, with input (blue) and output (red) polarizations mirrored about the equator. **b** Measured focal lengths for each polarization–wavelength channel. Error bars indicate deviation from the designed 700 μm focal plane. **c** Holographic patterns (target, simulation, and measurement) for five wavelength–polarization channels, demonstrating high fidelity and achromatic performance

To verify the feasibility of our proposed framework, we designed a non-degenerate metasurface hologram that maps a densely sampled continuous set of polarization states onto distinct wavelengths. As shown in Fig. 3a, the five channels are represented on the Poincaré sphere, where each colored ellipse denotes a specific elliptical polarization state assigned to a particular wavelength. The input (blue) and output (red) states are designed to be conjugate pairs, symmetrically mirrored about the equator to maximize birefringent contrast and satisfy polarization-path coupling conditions. These polarization–wavelength mappings are not arbitrarily selected, but rather aligned along a physically realizable, continuously evolving dispersion trajectory enabled by our dimension-interlaced design model.

The broadband achromatic performance is summarized in Fig. 3b, where focal length deviations across channels are quantitatively evaluated. All five channels converge near the 700 μm focal plane, with acceptable variation, even under practical fabrication and material dispersion conditions. The slight deviation at 3.055 μm is attributed to atmospheric absorption near water vapor resonance and its underweighted contribution in the optimization process.

The designed holographic patterns are shown in Fig. 3c for each channel, including the target, simulation, and measured results. All outputs exhibit high fidelity, with minimal distortion, defocus, or image degradation. These results confirm that our metasurface achieves robust achromatic holography across both spectral and polarization channels, even under non-degenerate conditions. Importantly, this result cannot be achieved using conventional spatial interleaving or focal-plane-based holography, which fundamentally rely on discrete channel separation and local DoF control. Here, we demonstrate for the first time that a single-layer metasurface with nonlocal design and continuous-domain reconstruction can simultaneously realize (i) multicolor channel isolation, (ii) polarization selectivity, and (iii) broadband achromatic focusing—all in a co-encoded fashion. The effectiveness of this approach stems from our precise co-optimization of intrinsic meta-atom dispersion and nonlocal field propagation, alongside our data-compressed neural modeling strategy, which allows high-dimensional polarization–wavelength control to be mapped into a realizable structural design.

**Fig. 4:**
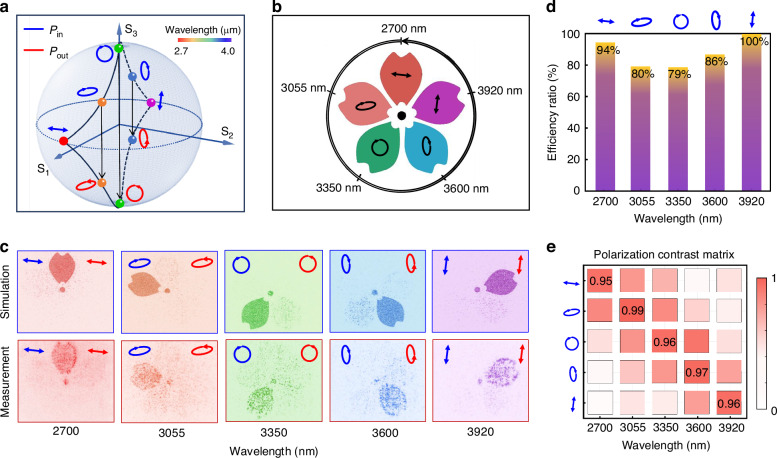
Characterization of arbitrary-polarization multicolor vector holography metasurface. **a** Arbitrary polarization states mapped to five representative wavelengths on the Poincaré sphere. Input (blue) and output (red) states are designed as conjugate pairs, mirrored about the equator. **b** Target vector holographic image, where each lobe encodes a unique wavelength–polarization channel. **c** Simulated and measured holographic reconstructions, demonstrating polarization–wavelength decoupling and inter-channel isolation. **d** Relative efficiency across the five vectorial channels, normalized by peak channel intensity. **e** Polarization contrast matrix, quantifying vectorial fidelity across designed polarization–wavelength states

To further validate the versatility of our theoretical framework, we extend the metasurface design to support arbitrary polarization dispersion paths beyond the linear trajectories. As illustrated in Fig. 4a, each wavelength is mapped to an elliptical polarization state selected freely on the Poincaré sphere, unconstrained by equatorial symmetry or linear interpolation. This represents a non-degenerate polarization–wavelength mapping, where input (blue) and output (red) polarization states form conjugate elliptical pairs, distributed arbitrarily across the polarization manifold.

The target holographic image in Fig. 4b consists of five spatially distinct lobes, each encoding a different polarization–wavelength channel. Achieving this arbitrary dispersion mapping poses significant challenges due to the need for precise, channel-specific birefringence control under a shared metasurface structure. Moreover, the orthogonality among channels must be maintained despite their non-monotonic dispersion behavior and abrupt variations in polarization state. Such requirements exceed the capability of conventional interleaved metasurfaces or focal-plane multiplexing methods, which rely on spatial segregation or linear dispersion approximations.

In Fig. 4c, both simulation and experimental results confirm the successful reconstruction of each vectorial channel with minimal distortion or crosstalk. The high isolation among channels is attributed to our dual-DoF engineering approach, which simultaneously exploits wavelength-selective and polarization-selective responses within the nonlocal metasurface design. To quantitatively assess the inter-channel uniformity, Fig. 4d presents the relative efficiency for each channel, normalized to the maximum measured value. The achieved balance across channels highlights the effectiveness of our design in equalizing energy distribution even under asymmetric dispersion conditions. And the nonlocal optimization ensures that all functional modes lie within the subwavelength scale, thereby eliminating higher-order leakage and confining optical energy to the 0th order, which defines the reconstructed holographic field. Slight deviations at 3.055 μm and 3.35 μm are primarily attributed to atmospheric absorption and etching-induced structural variations, respectively.

Furthermore, to evaluate the polarization conversion fidelity, we compute the polarization contrast (PC) matrix shown in Fig. 4e. This metric compares the measured polarization vector $|{\kappa }^{-} > ={\left[{A}_{1}{e}^{i{\delta }_{1}},{{A}}_{2}{e}^{i{\delta }_{2}}\right]}^{T}$ to the ideal target state ∣κ + 〉, using the contrast formula ${PC}={\left|{\left({\kappa }^{-}\right)}^{T}\cdot {\kappa }^{+}\right|}^{2}/\,{\left|{\kappa }^{+}\right|}^{2}$. The PC between target polarization channels exceeds 0.95 and low off-diagonal leakage confirm that the metasurface accurately distinguishes and reconstructs the intended elliptical states, even across varying wavelengths. The correlation coefficients and crosstalk metrics are provided in Supplementary Note 7. These quantitative results verify that the proposed nonlocal metasurface enables spectrally resolved and polarization-distinct reconstruction with minimal mutual interference. Additionally, although our experimental demonstration is limited to five discrete channels with ~200–300 nm channel spacing for visualization, the same nonlocal framework is scalable to tens or more polarization–wavelength states. Supplementary simulations confirm that by modestly relaxing isolation requirements, distinct holographic channels can be designed across sub-100 nm intervals, illustrating the pathway toward practically continuous modulation. Supporting simulations demonstrating these scalability pathways are provided in Supplementary Fig. S7.

It is worth noting that the current number of demonstrated channels is limited not by the physical framework as proved in previous content but by the finite sampling density of the meta-atom database. In principle, as the meta-atom library becomes denser, the neural network learns a higher-order interpolation between eigen-polarization and spectral response, enabling the design of densely spaced channels approaching a continuous mapping. We also provide a detailed discussion in the Supplementary Note 8 on the three key physical parameters (group-delay, eigen-polarizations, and spatial-frequency bandwidth) that fundamentally limit the infinite interpolation of holographic channels, along with an analysis of the challenges encountered in practical fabrication and optimization strategies.

**Fig. 5:**
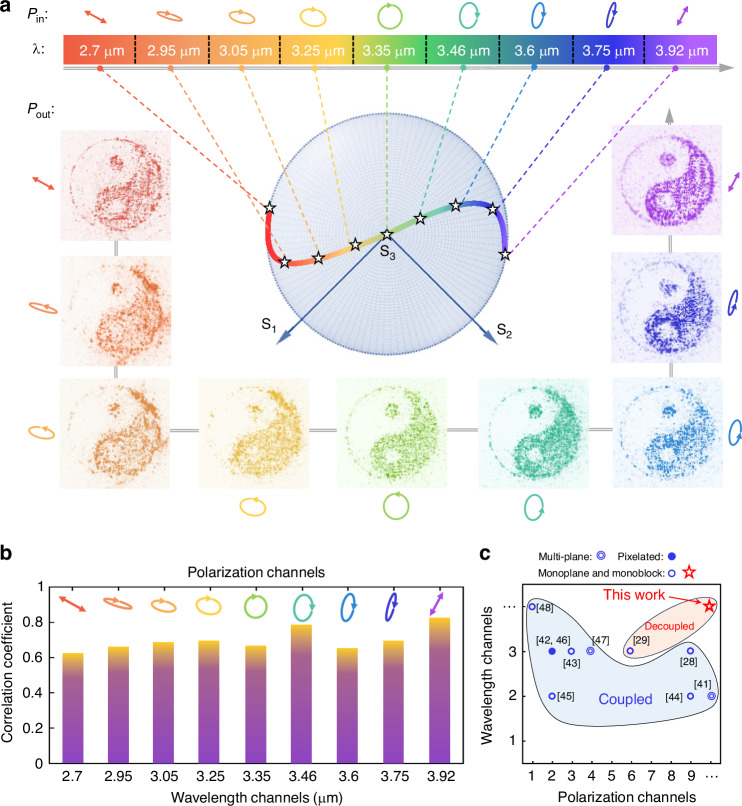
Characterization of continuous gradient-polarization holography across a broadband spectrum. **a** Broadband holography under arbitrarily varying linear polarization states from −30° to 60°, with conjugate-polarized input–output pairs mapped onto the Poincare sphere. The holography remains achromatic across 2700–3920 nm. **b** Correlation coefficients between experimentally reconstructed images and the target pattern across densely sampled continuous polarization and wavelength channels. **c** Comparison of polarization–wavelength channels achieved by our design versus previously reported metasurface holography works. Colored shading indicates whether polarization–wavelength decoupling was achieved

As shown in Fig. 5a, we design a densely sampled continuous polarization trajectory from −30° to 60° linear polarization, conjugated across the Poincaré sphere. This path is projected onto nine representative wavelengths spanning the mid-infrared regime (2700–3920 nm), under a numerical aperture of 0.43. The resulting vectorial hologram shows consistent high-fidelity reconstructions across both dimensions without image deformation or spatial scaling artifacts. To quantitatively assess the broadband and polarization robustness, Fig. 5b presents the correlation coefficients between experimental and target images across all sampled channels. The high correlation values confirm the device’s achromatic and vectorially adaptive behavior, validating the effectiveness of our compressive forward model under continuous dual-DoF variation. As previously mentioned, the analytical form (Eq. 4) allows smooth spectral–polarization evolution that can be densely sampled in both simulation and experiment to verify continuity. In Supplementary Note 9, we further performed dense interpolation simulations and supplementary experiments for the continuous-gradient achromatic hologram. The system was sampled at 1° polarization and 15 nm wavelength intervals. The simulated Stokes parameters exhibit monotonic evolution on the Poincaré sphere, confirming smooth polarization rotation with wavelength. Experimentally interpolated channels using additional band-pass filters show consistent behavior, validating that intermediate states are physically reconstructable rather than extrapolated. These results substantiate the concept of continuity as a smooth, differentiable mapping in the polarization–wavelength space.

In contrast to previous metasurface holography works—where broadband achromaticity was typically limited to circular polarization channels, or polarization multiplexing was achieved only via pixel-level interleaving—our platform offers fully continuous polarization tuning across a broad infrared spectral band within a single-layer, non-pixelated metastructure. The progress from discrete to continuous control is summarized in Fig. 5c, which benchmarks the polarization and wavelength channel control of our metadevice against leading reported designs. Colored regions highlight whether independent polarization–wavelength decoupling was achieved. Detailed quantitative comparisons of fidelity, crosstalk, and efficiency among different design methods are provided in the Supplementary Note 10. Our method uniquely supports both continuous-domain variation and decoupled encoding, distinguishing it from state-of-the-art alternatives. These results establish a metasurface platform capable of continuous gradient polarization control with broadband achromatic fidelity, setting the stage for high-dimensional information encoding, secure optical communication, and wavelength-adaptive imaging systems across diverse photonic applications. Additionally, by replacing Si with low-loss, high-index dielectrics and leveraging advanced high-aspect-ratio nanofabrication, our nonlocal metasurface design can be directly extended to the visible range. The key challenges lie in maintaining fabrication precision and suppressing scattering losses, both of which are being progressively addressed by recent developments in visible-band dielectric metasurface technology^16,50^.

## Discussion

Combining polarization and wavelength unlocks a vast encoding space for high-dimensional optics, yet practical metasurface implementations have remained constrained by intrinsic dispersion curvature and DoF entanglement. In this work, we establish a nonlocal metasurface framework that enables independent and densely sampled customize over polarization–wavelength states across a broadband spectrum. By introducing an equivalent nonlocal Jones matrix formalism and integrating a dimension-interlaced vectorial diffraction neural network, we achieve programmable vectorial holography beyond the discretized limits of conventional designs. Although independent phase modulation is implemented for different preset polarization states at a single wavelength, the continuous mapping relationship between different wavelengths and polarizations is deterministic, thereby enabling wavelength‑driven continuous polarization evolution. In practical applications, the holographic image and the output polarization states encoded thereon can be switched by varying the incident wavelength and the input polarization states. Our approach supports arbitrary elliptical polarization mapping, non-degenerate multicolor holography, and achromatic operation from 2.7 to 4 μm. These results demonstrate a shift from static DoF segmentation to continuous-domain optical field design, offering a scalable foundation for high-capacity light-field encoding, reconfigurable metasystems, and next-generation vectorial communication technologies.

## Materials and methods

### Sample fabrication and details

The samples were fabricated on a double-side polished silicon wafer. A 100 nm-thick chromium layer was first deposited via electron-beam evaporation. Negative photoresist was spin-coated at 3000 rpm for 30 s and soft-baked. The patterns were exposed using JEOL electron-beam lithography. After exposure, the sample was developed, rinsed and dried. The silicon was subsequently etched using inductively coupled plasma. The process achieved a minimum feature size of 300 nm and a minimum inter-feature spacing of 350 nm, enabling the fabrication of high-aspect-ratio (∼20:1), 7 μm-deep nanopillars essential for broadband birefringent dispersion control. The fabricated device occupies a 600 × 600 μm area and comprises a 400 × 400 meta-unit array, with a designed focal plane at 700 μm. Each of the five operating wavelengths (2.7 μm, 3.055 μm, 3.35 μm, 3.6 μm, and 3.92 μm) is mapped to a unique polarization channel characterized by principal axis angles from 0 to π/2, under a fixed phase retardation Δφ = π/2, thereby satisfying the conjugate elliptical polarization modulation condition derived in Eq. (4).

### Measurement procedure

The experimental characterization setup consisted of six primary components: (i) a broadband laser source, (ii) wavelength filters, (iii) polarization generation, (iv) the metadevice under test, (v) polarization analysis, and (vi) the imaging module. A supercontinuum mid-infrared laser (Electro-MIR) served as the broadband source. Nine bandpass filters (Thorlabs and Edmund Optics; FB-2700-110 nm, FB-2950-110 nm, FB-3055-155 nm, FB-3250-500 nm, FB-3350-120 nm, FB-3460-140 nm, FB-3600-140 nm, FB-3750-200 nm, FB-3920-179 nm) were used to select discrete wavelengths across the 2.7–3.9 μm spectral range. Polarization states were generated and analyzed using linear polarizers and liquid-crystal variable retarders (LCC1113-MIR). The optical response was captured using a mid-infrared imaging system consisting of a 4 mm aspheric lens, a 25 mm relay lens, and a mercury cadmium telluride (MCT) focal-plane-array camera.

### Metasurface design and simulation

The numerical simulations were conducted using the finite-difference time-domain (FDTD) method. Periodic boundary conditions were applied along the x and y directions, and perfectly matched layers (PMLs) were used along the z direction to prevent nonphysical reflections. The metasurface unit cell consisted of a silicon nanopillar with a height of 7 μm and a period of 1.5 μm. To construct the design library, the phase and transmission responses under x- and y-polarized incident light were calculated by sweeping the pillar length and width from 350 to 1450 nm in 10 nm increments. The illumination was introduced as a normally incident plane wave from the substrate side. Here, a dimension-interlaced vectorial-diffraction neural network (DVNN) was developed in a Python v3.8.13 and PyTorch v1.12.1 (Facebook Inc.) to design the hologram by embedding our compressed analytical model within a differentiable pipeline. Each meta-atom neuron is defined by its complex Jones matrix, and the forward–backward propagation follows the vectorial diffraction equation (Eq. 4) rather than stochastic sampling of a dataset. The equivalent nonlocal Jones matrix at the observation plane is reconstructed by solving for a global diffraction field that satisfies both the spectral and polarization constraints, with polynomial coefficients optimized by Adam. The algorithm does not rely on external training data, the effective “dataset” corresponds to the meta-atom library used to parameterize the Jones matrix space. The continuous-dimension overlap formalism compresses the computational complexity from 4n^3^ to 8n, ensuring convergence and scalability with negligible accuracy loss.

## Supplementary information

Supplementary Material

## Data Availability

Relevant data supporting the key findings of this study are available in the article and Supplementary Information file. All raw data generated in this study are available from the corresponding authors upon reasonable request.

## References

[1] Zhang, L. J., Silberhorn, C. & Walmsley, I. A. Secure quantum key distribution using continuous variables of single photons. *Phys. Rev. Lett.* **100**, 110504 (2008). 18517770 10.1103/PhysRevLett.100.110504

[2] Cui, K. Y. et al. Spectral convolutional neural network chip for in-sensor edge computing of incoherent natural light. *Nat. Commun.* **16**, 81 (2025). 39747892 10.1038/s41467-024-55558-3 PMC11696300

[3] Lo, H. K., Curty, M. & Tamaki, K. Secure quantum key distribution. *Nat. Photonics* **8**, 595–604 (2014).

[4] Jouguet, P. et al. Experimental demonstration of long-distance continuous-variable quantum key distribution. *Nat. Photonics* **7**, 378–381 (2013).

[5] Zhang, Z. et al. Structured light meets machine intelligence. *eLight* **5**, 26 (2025).

[6] You, C. & Magaña-Loaiza, O. S. Chip-based quantum signature network reaches 200 km. *Light Adv. Manuf.* **6**, 4 (2025).

[7] Yuan, H. et al. Multi-channel image encryption based on an all-dielectric metasurface incorporating near-field nanoprinting and far-field holography. *Adv. Opt. Mater.* **11**, 2300352 (2023). 10.1364/OE.505549 38178477

[8] Wan, W. P. et al. Tunable full-color vectorial meta-holography. *Adv. Opt. Mater.* **10**, 2201478 (2022).

[9] Liu, M. Z. et al. Multifunctional metasurfaces enabled by simultaneous and independent control of phase and amplitude for orthogonal polarization states. *Light Sci. Appl.* **10**, 107 (2021). 34035215 10.1038/s41377-021-00552-3 PMC8149653

[10] So, S. et al. Multicolor and 3D holography generated by inverse-designed single-cell metasurfaces. *Adv. Mater.* **35**, 2208520 (2023). 10.1002/adma.202208520 36575136

[11] Thiele, S. et al. 3D-printed eagle eye: Compound microlens system for foveated imaging. *Sci. Adv.* **3**, e1602655 (2017). 28246646 10.1126/sciadv.1602655 PMC5310822

[12] Yu, N. F. et al. Light propagation with phase discontinuities: generalized laws of reflection and refraction. *Science* **334**, 333–337 (2011). 21885733 10.1126/science.1210713

[13] Rubin, N. A. et al. Matrix Fourier optics enables a compact full-Stokes polarization camera. *Science* **365**, eaax1839 (2019). 31273096 10.1126/science.aax1839

[14] Yang, Y. et al. Integrated metasurfaces for re-envisioning a nearfuture disruptive optical platform. *Light Sci. Appl.* **12**, 152 (2023). 37339970 10.1038/s41377-023-01169-4 PMC10282098

[15] Ren, H. R. et al. Complex-amplitude metasurface-based orbital angular momentum holography in momentum space. *Nat. Nanotechnol.* **15**, 948–955 (2020). 32958936 10.1038/s41565-020-0768-4

[16] Wang, Y. J. et al. High-efficiency broadband achromatic metalens for near-IR biological imaging window. *Nat. Commun.* **12**, 5560 (2021). 34548490 10.1038/s41467-021-25797-9 PMC8455568

[17] Chen, S. Q. et al. Metasurface-empowered optical multiplexing and multifunction. *Adv. Mater.* **32**, 1805912 (2020). 10.1002/adma.201805912 31617616

[18] Arbabi, A. et al. Dielectric metasurfaces for complete control of phase and polarization with subwavelength spatial resolution and high transmission. *Nat. Nanotechnol.* **10**, 937–943 (2015). 26322944 10.1038/nnano.2015.186

[19] Zhou, Y. et al. Meta-device for field-of-view tunability via adaptive optical spatial differentiation. *Adv. Sci.* **12**, 2412794 (2025). 10.1002/advs.202412794 PMC11884584 39806861

[20] Leng, B., Zhang, Y., Tsai, D. P. & Xiao, S. Meta-device: advanced manufacturing. *Light Adv. Manuf.* **5**, 5 (2024).

[21] Song, Y. Z. et al. Three-dimensional varifocal meta-device for augmented reality display. *PhotoniX.* **6**, 6 (2025).

[22] Cheng, J. L. et al. Tunable meta-device for large depth of field quantitative phase imaging. *Nanophotonics.* **14**, 1249–1256 (2025). 40290285 10.1515/nanoph-2024-0661 PMC12019949

[23] Cheng, J. L. et al. Quantitative phase imaging for meta-lenses by phase retrieval. *Adv. Opt. Mater.* **13**, 2402833 (2025).

[24] Rubin, N. A. et al. Jones matrix holography with metasurfaces. *Sci. Adv.* **7**, eabg7488 (2021). 34389537 10.1126/sciadv.abg7488 PMC8363145

[25] Deng, Z. L. et al. Diatomic metasurface for vectorial holography. *Nano Lett.* **18**, 2885–2892 (2018). 29590530 10.1021/acs.nanolett.8b00047

[26] Yang, H. et al. Noninterleaved metasurface for full-polarization three-dimensional vectorial holography. *Laser Photonics Rev.* **16**, 2200351 (2022).

[27] Xiong, B. et al. Breaking the limitation of polarization multiplexing in optical metasurfaces with engineered noise. *Science* **379**, 294–299 (2023). 36656947 10.1126/science.ade5140

[28] Wang, J. et al. Unlocking ultra-high holographic information capacity through nonorthogonal polarization multiplexing. *Nat. Commun.* **15**, 6284 (2024). 39060283 10.1038/s41467-024-50586-5 PMC11282074

[29] Chen, J. et al. Polychromatic full-polarization control in mid-infrared light. *Light Sci. Appl.* **12**, 105 (2023). 37142624 10.1038/s41377-023-01140-3 PMC10160079

[30] Shi, Z. J. et al. Continuous angle-tunable birefringence with freeform metasurfaces for arbitrary polarization conversion. *Sci. Adv.* **6**, eaba3367 (2020). 32537506 10.1126/sciadv.aba3367 PMC7269657

[31] Xie, X. et al. Generalized Pancharatnam-Berry phase in rotationally symmetric meta-atoms. *Phys. Rev. Lett.* **126**, 183902 (2021). 34018802 10.1103/PhysRevLett.126.183902

[32] Balthasar Mueller, J. P. et al. Metasurface polarization optics: independent phase control of arbitrary orthogonal states of polarization. *Phys. Rev. Lett.* **118**, 113901 (2017). 28368630 10.1103/PhysRevLett.118.113901

[33] Overvig, A. C. et al. Dielectric metasurfaces for complete and independent control of the optical amplitude and phase. *Light Sci. Appl.* **8**, 92 (2019). 31666948 10.1038/s41377-019-0201-7 PMC6804926

[34] Abdollahramezani, S. et al. Electrically driven reprogrammable phase-change metasurface reaching 80% efficiency. *Nat. Commun.* **13**, 1696 (2022). 35354813 10.1038/s41467-022-29374-6 PMC8967895

[35] Wu, X. et al. A multi-resonant tunable Fabry-Pérot cavity for high throughput spectral imaging. *Adv. Opt. Mater.* **13**, 2402784 (2025).

[36] de Galarreta, C. R. et al. Reconfigurable multilevel control of hybrid all-dielectric phase-change metasurfaces. *Optica.* **7**, 476–484 (2020).

[37] Han, F. et al. Tunable mid-infrared multi-resonant graphene-metal hybrid metasurfaces. *Adv. Opt. Mater.* **12**, 2303085 (2024).

[38] Lawrence, M. et al. High quality factor phase gradient metasurfaces. *Nat. Nanotechnol.* **15**, 956–961 (2020). 32807879 10.1038/s41565-020-0754-x

[39] Zeng, Y. X. et al. Metalasers with arbitrarily shaped wavefront. *Nature* **643**, 1240–1245 (2025). 40634607 10.1038/s41586-025-09275-6

[40] Liang, Y., Tsai, D. P. & Kivshar, Y. From local to nonlocal high-*Q* plasmonic metasurfaces. *Phys. Rev. Lett.* **133**, 053801 (2024). 39159090 10.1103/PhysRevLett.133.053801

[41] Intaravanne, Y. et al. Creating wavelength-selective polarization digital numbers. *Adv. Opt. Mater.* **12**, 2203097 (2024).

[42] Jin, L. et al. Noninterleaved metasurface for (2^6^-1) spin- and wavelength-encoded holograms. *Nano Lett.* **18**, 8016–8024 (2018). 30520648 10.1021/acs.nanolett.8b04246

[43] Hu, Y. Q. et al. Trichromatic and tripolarization-channel holography with noninterleaved dielectric metasurface. *Nano Lett.* **20**, 994–1002 (2019). 10.1021/acs.nanolett.9b04107 31880939

[44] Kim, J. et al. Photonic encryption platform *via* dual-band vectorial metaholograms in the ultraviolet and visible. *ACS Nano.* **16**, 3546–3553 (2022). 35184548 10.1021/acsnano.1c10100

[45] Wang, B. et al. Polarization-controlled color-tunable holograms with dielectric metasurfaces. *Optica.* **4**, 1368–1371 (2017).

[46] Yin, Y. Y. et al. Multi-dimensional multiplexed metasurface holography by inverse design. *Adv. Mater.* **36**, 2312303 (2024). 10.1002/adma.202312303 38372628

[47] Guo, X. Y. et al. Full-color holographic display and encryption with full-polarization degree of freedom. *Adv. Mater.* **34**, 2103192 (2022). 10.1002/adma.202103192 34363242

[48] Song, Q. et al. Bandwidth-unlimited polarization-maintaining metasurfaces. *Sci. Adv.* **7**, eabe1112 (2021). 33514552 10.1126/sciadv.abe1112 PMC7846164

[49] Zhao, R. Z. et al. Multichannel vectorial holographic display and encryption. *Light Sci. Appl.* **7**, 95 (2018). 30510691 10.1038/s41377-018-0091-0 PMC6258690

[50] Wang, S. M. et al. A broadband achromatic metalens in the visible. *Nat. Nanotechnol.* **13**, 227–232 (2018). 29379204 10.1038/s41565-017-0052-4

