# Supplementary material for: Continuous polarization–wavelength mapping with nonlocal metasurfaces
Source: Light Sci Appl. 2026 Mar 13;15:170. doi: 10.1038/s41377-026-02233-5 (PMC12982823; doi:10.1038/s41377-026-02233-5)
Supplement: Supplementary file 1 — Supplementary Material [file 41377_2026_2233_MOESM1_ESM.pdf]

Supplementary Materials for:

Continuous Polarization–Wavelength Mapping with Nonlocal Metasurfaces

Jiuxu Wang[1,2#], Jie Wang[1#], Feilong Yu[1#*], Jin Chen[1], Rongsheng Chen[1], Tianxiong Geng[1], Rong Jin[1,2], Yiran Zhou[1], Tongwen Zheng[1], Guanhai Li[1,2,3,4]*, Xiaoshuang Chen[1,2,3,4*], and Wei Lu[1,2,3,4*]

[1]*State Key Laboratory of Infrared Physics, Shanghai Institute of Technical Physics, Chinese Academy of Sciences, 500 Yu-Tian Road, Shanghai, 200083, China*

[2]*University of Chinese Academy of Sciences, No.19 Yuquan Road, Beijing 100049, China*

[3]*Hangzhou Institute for Advanced Study, University of Chinese Academy of Sciences, No.1 SubLane Xiangshan, Hangzhou, 310024, China*

[4]*Shanghai Research Center for Quantum Sciences, 99 Xiupu Road, Shanghai, 201315, China*

*ghli0120@mail.sitp.ac.cn; xschen@mail.sitp.ac.cn; yufeilong@mail.sitp.ac.cn luwei@mail.sitp.ac.cn

Note 1. Decouple the continuously conjugated polarization channels from spectral dispersion

For the fast and slow axis-symmetric linearly birefringent elements, the precession axis of polarization states conversion is located at the equator of the Poincaré sphere and determined by the angular orientation of elements. Only the input and output polarization states are mirrored about the equator or conjugate, the rotation angle DoF of elements can be harnessed for precise phase control. Therefore, the conjugate channel flipped handedness between the input and output polarization is essential for continuous-domain photonic multiplexing. Unlike traditional broadband achromatic designs that are limited to circular polarization channels, the proposed continuous information-domain reconstruction framework is broadly applicable to arbitrary conjugate elliptical polarization channels. The comparison of the polarization evolutions between circular and general elliptical polarization is shown on the Poincaré sphere in Figure S1, where the shaded regions indicate the variation in the rotation angle of meta-atom elements.

For traditional polarization-wavelength-degenerate elements structure, where the phase retardation φ_β - φ_α of Y polarization and the X polarization component is independent of wavelength as the inset shows, the conjugate modulation phase (CMP, red line) varies linearly with the rotation angle θ as shown in the left panel of Figure S1. The birefringence phase retardation (BPR, blue line) always remains π, consistent with a half-wave plate. In contrast, for birefringent dispersion elements, CMP exhibits a nonlinear dependence on θ and the corresponding BPR requirement for each θ is no longer fixed as shown in the right panel of Figure S1. Due to θ being invariant across the metasurfaces array, the function relationship between BPR and θ varies with different elliptical polarization conjugated channels. This implies that an additional DoF, i.e., wavelength, is necessary to decouple the continuous polarization evolution. Moreover, θ can be treated as a perturbation parameter to optimize the far-field information within the continuous polarization-wavelength domain. Within our design framework, the initial step involves defining the range of rotational angle perturbations and establishing the initial iteration value. This process is guided by the projection of the continuous polarization channel, in conjunction with a third-order or higher-order

nonlinear approximation of the unit dispersion response. Subsequently, a hierarchical, dimension-interlaced vectorial diffraction neural network is employed to further refine the solution, with the goal of achieving a global optimum via nonlocal Pancharatnam–Berry phase evolution, as elaborated in the main manuscript.

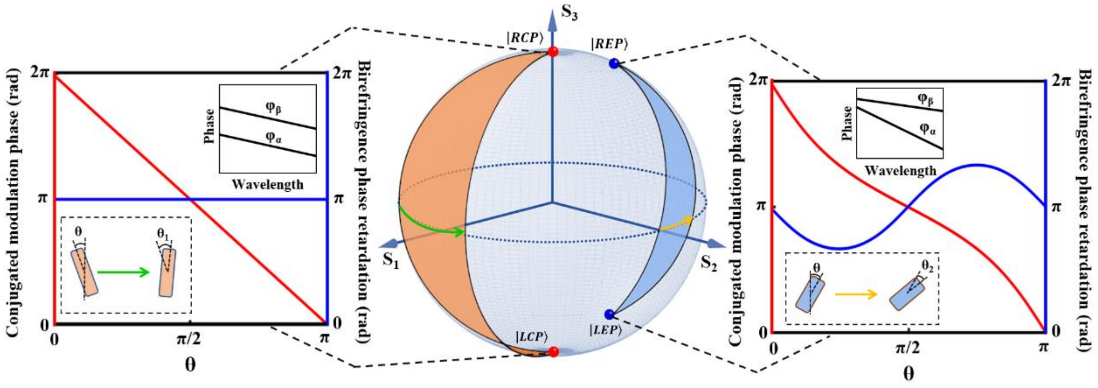

Fig. S1. Comparison between arbitrary elliptical polarization modulation (right) and conventional broadband circular polarization modulation (left). Left: For circular polarization state, polarization–wavelength-degenerate elements exhibit a constant birefringent phase retardation of π (blue line), while the conjugate modulation phase (red line) varies linearly with twice the element rotation angle θ. Right: For arbitrary elliptical polarization state, the conjugate modulation phase exhibits a nonlinear dependence on the element rotation angle, and the birefringent retardation varies with the rotation angle, rather than remaining fixed. This general case necessitates the introduction of a dispersion-compensating element (inset). Center: Polarization evolution on the Poincaré sphere, where the shaded regions indicate the variation in the polarization precession axis associated with different element rotation angles.

Note 2. Physical mechanism of the nonlocal Jones matrix decoupling continuous polarization–wavelength channels

2.1 Limitations of conventional light-manipulation principles

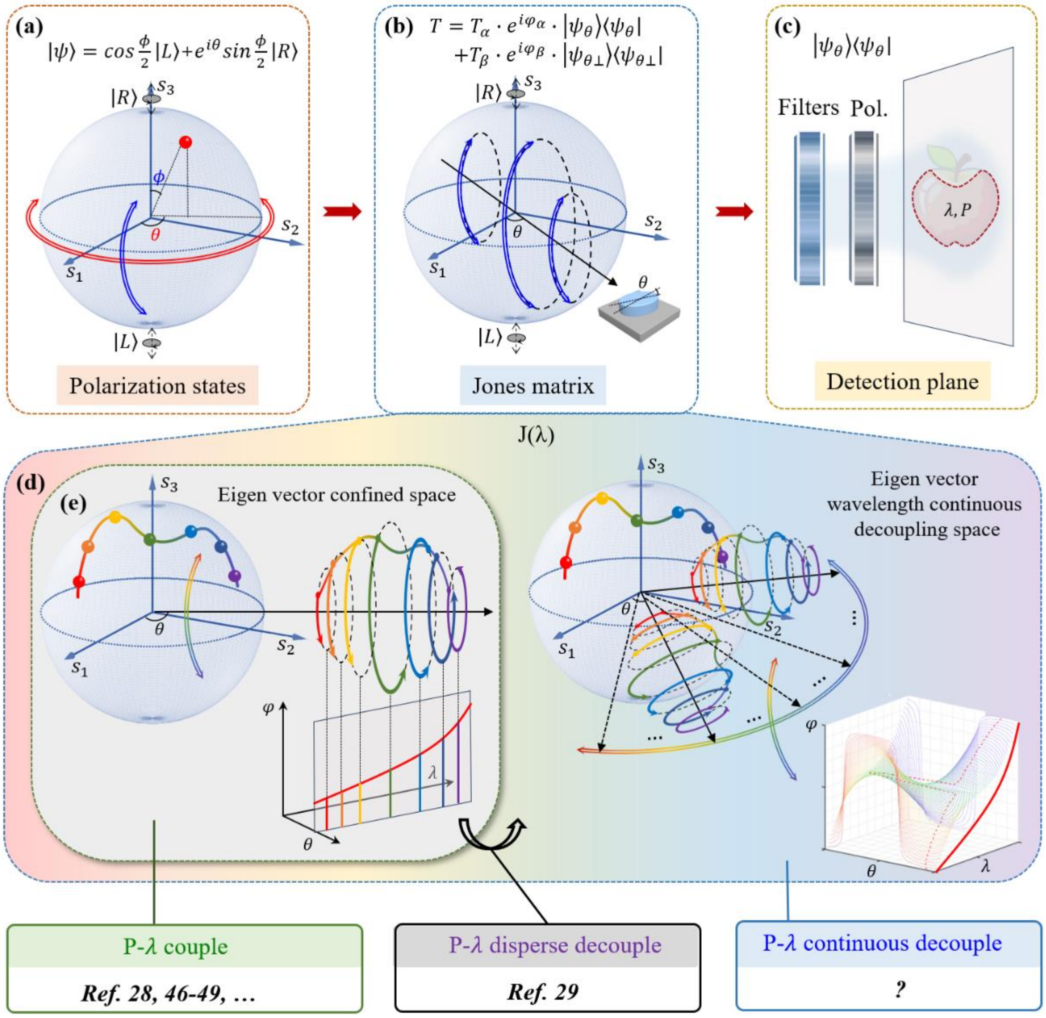

Fig. S2. The physical process of photon control and detection. (a) The polarization state in the circular polarization basis vectors. (b) Geometrical dependence of the metaatom on the control basis vectors. (c) Polarization–wavelength information on the detection plane. (d) Continuous polarization–wavelength control space constructed by the Jones matrix incorporating the wavelength. (e) The eigen-basis vector confined space.

The photonic polarization state is expressed as a superposition of the complex amplitudes of two orthogonal circular polarization basis vectors, as shown on the Poincaré sphere in Fig. S2(a):

$$|\psi\rangle = cos\frac{\phi}{2}\cdot|L\rangle + e^{i\theta}\cdot sin\frac{\phi}{2}|R\rangle \qquad (S1)$$

During the microstructural modulation process, the geometric characteristics of the structure determine its symmetry modal, thereby defining the basis vectors for

manipulating the Jones matrix. Consequently, polarization states that are originally equivalent under arbitrary decompositions in the state space become constrained to specific basis evolution paths within the structure. For a structure with a rotation angle of θ:

$$T = T_\alpha \cdot e^{i\varphi_\alpha} \cdot |\psi_\theta\rangle\langle\psi_\theta| + T_\beta \cdot e^{i\varphi_\beta} \cdot |\psi_{\theta\perp}\rangle\langle\psi_{\theta\perp}| \tag{S2}$$

T_α and T_β regulate the amplitude ratio between two orthogonal basis vectors with the modulation path aligned along the axes defined by the basis directions. φ_α and φ_β control the phase difference between the orthogonal basis vectors, with the modulation path corresponding to the tangential direction of concentric circles centered on the basis vector axes. For a single structural element, although dispersion may alter the mode modulation, the symmetry-related modulation basis vectors remain unchanged, exhibiting wavelength degeneracy of the eigen-polarizations. As shown in Fig. S2(b), the constraint of the modulation path on the Poincaré sphere limits the ability to achieve arbitrary polarization control. In existing metasurface-based optical modulation, the phase dispersions along the two modulation basis vectors are approximately linear of the primary resonance mode, and can be expressed as:

$$\begin{aligned} \varphi_\alpha &\approx k_1\lambda + b_1 \\ \varphi_\beta &\approx k_2\lambda + b_2 \end{aligned} \tag{S3}$$

The regulation behavior of the photon state is shown in Fig. S2(e). The geometric dependence of the controlled basis vectors constrains the modulation trajectory, so that the accessible paths occupy only a portion of the entire Poincaré sphere. In other words, the polarization basis and wavelength degeneracy impose intrinsic limitations on the range of photon information modulation supported by the structure. Most existing works, such as Ref. 25, 43-46 mentioned in the main text, achieve modulation mechanisms restricted to this confined region. Only a few studies have realized limited and irregular polarization–dispersion modulation under discrete wavelength conditions, typically by combining different metaatoms and employing approximate fitting strategies. For example, in our previous work Ref. 26, this limitation of the modulation space was partially overcome at discrete physical distances. However, no work has achieved a truly continuous and complete photon information modulation range along the wavelength dimension so far. This imposes a fundamental constraint on arbitrary polarization–wavelength manipulation. In the case of fully conjugate polarization–

dispersion holographic control, the structural modulation requirement can be directly expressed as (see Supplementary Note 3 for details):

$$tan\left(-\frac{\Delta\varphi(\lambda)}{2}\right) = \frac{tan(2P_1(\lambda))}{sin2(P_2(\lambda)+\theta(\lambda))} \quad (S4)$$

This not only requires a relatively complex phase-dispersion profile but also demands that the rotation angle of the eigen-basis be tunable with wavelength. Such functionality cannot be achieved within the conventional modulation space. The present work focuses on breaking this fundamental limitation of light manipulation, enabling wavelength-tunable control over the eigen-basis evolution path, thereby activating the full photon control space and ultimately realizing arbitrary manipulation of the polarization and wavelength of photons.

2.2 Breaking the limitation of local modulation via nonlocal Jones matrix

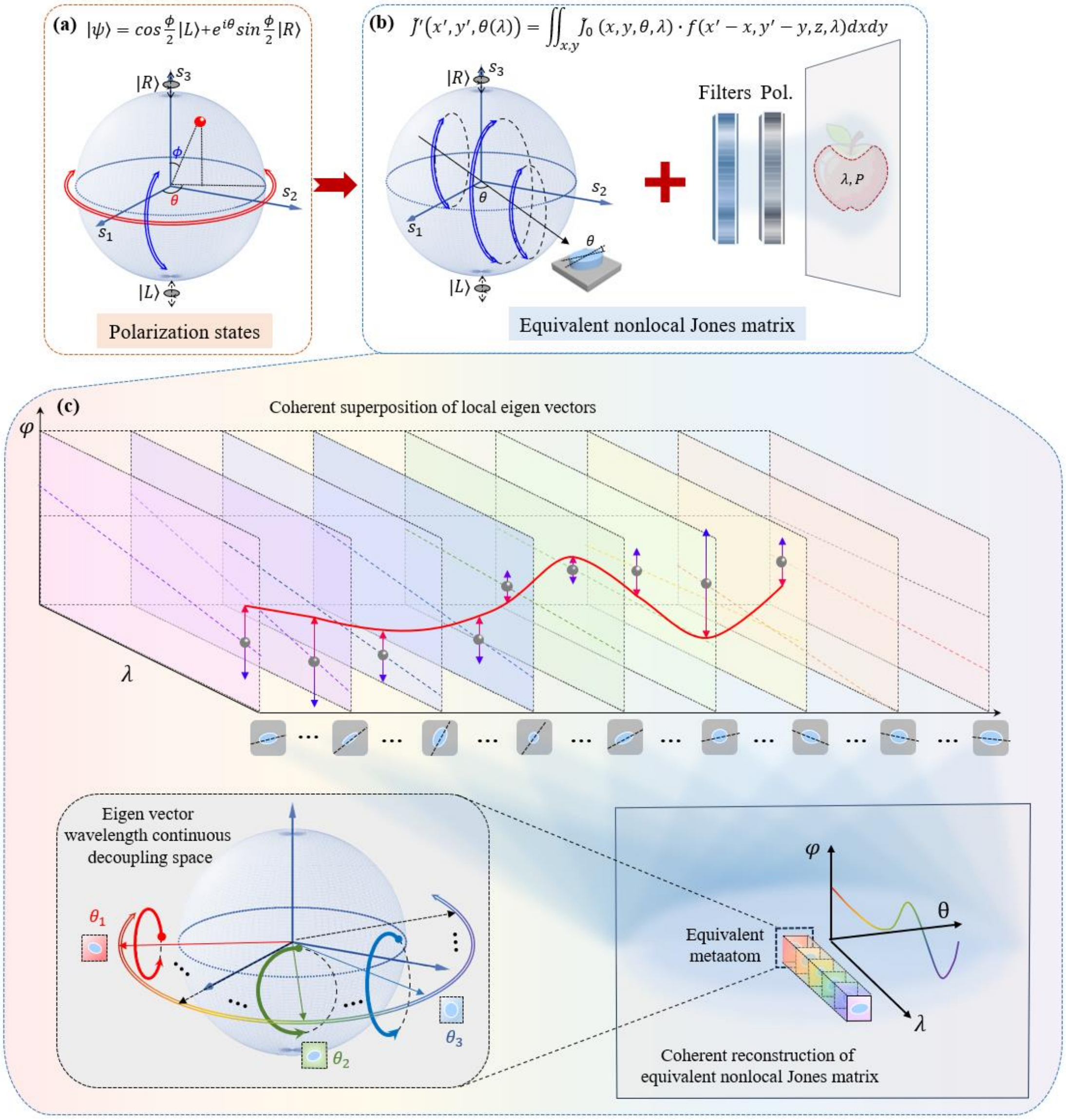

Fig. S3. Far-field reconstruction of the equivalent nonlocal Jones matrix. (a) The polarization state in the circular polarization basis. (b) Equivalent nonlocal Jones matrix constructed by combining local eigenmode modulation with coherent far-field diffraction superposition. (c) Reconstruction process of the equivalent geometric phase with tunable dispersion characteristics.

The proposed nonlocal Jones matrix optical field reconstruction theory, applicable under continuous wavelength and polarization conditions, is established on the fundamental concept that the modulation information carried by photons can be arbitrarily reconstructed at any position before the detector. This enables the transfer of photon modulation design from the local physical control plane to the detection plane, achieving a global equivalent reconstruction, as shown in Fig. S3(b). The process of nonlocal far-field coherent reconstruction can be expressed as:

$$\tilde{J}'\left(x', y', \theta(\lambda)\right) = \iint_{x,y} t_0(\lambda) \cdot e^{i\varphi_0(\lambda)} \cdot R\left(-\theta_{\left(P(\lambda),\Delta\varphi(\lambda)\right)}\right) \cdot \begin{pmatrix} 1 & 0 \\ 0 & e^{i\Delta\varphi(\lambda)} \end{pmatrix} \cdot R\left(\theta_{\left(P(\lambda),\Delta\varphi(\lambda)\right)}\right) \cdot f(x'-x, y'-y, z, \lambda)dxdy \quad (S5)$$

The underlying physical process can be described as follows: by locally tailoring the intrinsic polarization-dispersion response on the modulation surface and utilizing the far-field diffraction-based weighting mechanism of a neural network, a nonlocal coherent reconstruction is achieved on the observation plane, giving rise to a novel equivalent Jones matrix with tunable dispersive geometric characteristics. This equivalent nonlocal Jones matrix exhibits wavelength-tunable symmetry angles and control basis vectors, as well as high-order phase dispersion curves. As shown in Fig. S3(c), we present the physical process whereby a series of metaatoms—subject to quasi-linear phase dispersion and dispersion-bounded basis vectors—are coherently reconstructed through far-field diffraction superposition into an equivalent unit that possesses wavelength-tunable symmetric angles, adjustable control bases, and high-order phase dispersion characteristics.

In this case, the equivalent modulation unit does not correspond one-to-one with a specific physical structure, but rather features a tunable dispersive rotation angle, thereby breaking the conventional symmetry constraints of structural design. This mechanism effectively activates the dispersion control space that is restricted in traditional systems and combines it with the inherent phase degrees of freedom, thereby expanding a continuously accessible photonic control space across a broad wavelength

range. Consequently, continuous and arbitrary control over the photon polarization–wavelength relationship can be achieved.

2.3 Design method for nonlocal Jones matrix modulation

In terms of implementation, the proposed reconstruction based on geometric symmetry essentially controls the coherent superposition of geometric phases within the Jones matrix. This approach overcomes the computational bottleneck associated with conventional reconstruction under linear polarization bases, where the eigenvectors of the Jones matrix must be solved individually. Theoretically, it extends the controllable space of photonic information from a finite to a high-dimensional information space. In the circular polarization basis representation, the inverse-solving process of the Jones matrix is transformed into a direct coherent superposition of geometric phases. By directly manipulating the contributions of structural parameters to the geometric phase dispersion in the reconstructed modulation operator, we achieve the reconstruction of geometric rotation angles:

$$\boldsymbol{J}\,(\lambda) = \iint_{xy} \boldsymbol{J_{cir}}\,(\lambda) \cdot f(x' - x, y' - y, z, \lambda)dxdy$$

$$\boldsymbol{J_{cir}}\,(\lambda) = \left(\frac{\sqrt{2}}{2}\begin{pmatrix} 1 & 1 \\ i & -i \end{pmatrix}\right)^{-1} \cdot \begin{pmatrix} cos\alpha_\lambda & -\sin\alpha_\lambda e^{i\beta_\lambda} \\ \sin\alpha_\lambda e^{i\beta_\lambda} & cos\alpha_\lambda \end{pmatrix} \cdot$$

$$\begin{pmatrix} t_\alpha(\lambda) \cdot e^{i\varphi_\alpha(\lambda)} & 0 \\ 0 & t_\beta(\lambda) \cdot e^{i\varphi_\beta(\lambda)} \end{pmatrix} \cdot \begin{pmatrix} cos\alpha_\lambda & \sin\alpha_\lambda e^{i\beta_\lambda} \\ -\sin\alpha_\lambda e^{i\beta_\lambda} & cos\alpha_\lambda \end{pmatrix} \cdot \frac{\sqrt{2}}{2}\begin{pmatrix} 1 & 1 \\ i & -i \end{pmatrix}$$

$$= \begin{pmatrix} A & \mathrm{B} * e^{i\varphi_{b\lambda}} \\ \mathrm{C} * e^{i\varphi_{c\lambda}} & A \end{pmatrix} \qquad (S6)$$

Subsequently, the physical mechanism was incorporated into a diffraction neural network framework featuring a continuous wavelength dimension. By introducing a data-space compression mechanism under the conjugate-control requirement, global reconstruction across a continuous wavelength range becomes feasible even under constrained computational resources (Supplementary Note 5). Consequently, arbitrary control of photon-conjugated polarization and wavelength is realized.

Note 3. The forward analytical model for continuous polarization-wavelength path

The interaction between light and optical structures can be described using a 2×2 Jones matrix ***J*** (x, y) and corresponding Jones vectors. The matrix ***J*** (x, y) characterizes the optical response of individual metaatoms at each spatial coordinate (x, y), comprising four complex elements—equivalently, eight independent parameters, including both amplitude and phase. Consequently, these eight DoFs can, in principle, be fully controlled to realize completely independent optical functions without relying on additional physical dimensions. In the case of C_2-symmetrical metaatoms:

$$\boldsymbol{J}\,(x,y) = R\big(-\theta(\lambda)\big)\cdot\begin{pmatrix} t_{xx} & 0 \\ 0 & t_{yy}\end{pmatrix}\cdot R\big(\theta(\lambda)\big) \qquad (S7)$$

where $R(\theta) = \begin{bmatrix} cos\theta & sin\theta \\ -sin\theta & cos\theta \end{bmatrix}$ is the rotation matrix of the metaatoms; t_{xx} and t_{yy} are complex transmittance coefficients. The input polarization state can also be described with a rotation matrix and ellipticity angle $P_1(\lambda)$, $\tilde{P} = R(-P_2(\lambda))\cdot \begin{pmatrix} cosP_1(\lambda) \\ i\cdot sinP_1(\lambda)\end{pmatrix}$. To distinguish it from the rotation angle θ of the metaatoms, we denote the counterclockwise rotation angle of the input polarization as $P_2(\lambda)$. With phase modulation provided by the metaatoms—while maintaining approximately equal amplitude transmission coefficients $|t_{xx}|$=$|t_{yy}|$—the resulting output polarization state can be expressed as:

$$\begin{aligned}\widetilde{\mathrm{P}^{-}} &= \boldsymbol{J}\,(x,y)\cdot R\big(-P_2(\lambda)\big)\cdot\begin{pmatrix} cosP_1(\lambda) \\ i\cdot sinP_1(\lambda)\end{pmatrix} \\ &= R\big(-\theta(\lambda)\big)\cdot\begin{pmatrix} e^{i\varphi_\alpha(\lambda)} & 0 \\ 0 & e^{i\varphi_\beta(\lambda)}\end{pmatrix}\cdot R\big(\theta(\lambda)\big)\cdot R\big(-P_2(\lambda)\big)\cdot\begin{pmatrix} cosP_1(\lambda) \\ i\cdot sinP_1(\lambda)\end{pmatrix} \qquad (S8)\end{aligned}$$

By incorporating the rotation matrix, the output polarization state can be expressed as:

$$\widetilde{\mathrm{P}^{-}} = \begin{pmatrix} e^{i\varphi_\alpha(\lambda)} & 0 \\ 0 & e^{i\varphi_\beta(\lambda)}\end{pmatrix}\cdot R\big(-P_1(\lambda)-\theta(\lambda)\big)\cdot\begin{pmatrix} cosP_1(\lambda) \\ i\cdot sinP_1(\lambda)\end{pmatrix} \qquad (S9)$$

This implies that the eigenvector transformation of a birefringent metaatom has its eigen-polarization precession axis fixed on the equator of the Poincaré sphere, determined solely by the metaatom's rotation angle θ. To enable the engineering of

continuous polarization states, additional DoFs must be introduced to decouple the continuous polarization channels, such as the wavelength dimension. Thus, the dispersive Jones matrix can be written as

$$\boldsymbol{J}\,(x,y,\lambda)=R\big(-\theta(\lambda)\big)\cdot\begin{pmatrix}e^{i\varphi_\alpha(\lambda)} & 0\\ 0 & e^{i\varphi_\beta(\lambda)}\end{pmatrix}\cdot R\big(\theta(\lambda)\big)\qquad(S10)$$

Here, the continuous polarization channels vary with the continuous wavelengths. The input polarization states are represented in the form:

$$\widetilde{\mathrm{P}^{+}}=R\big(-P_2(\lambda)\big)\cdot\begin{pmatrix}cosP_1(\lambda)\\ i\cdot sinP_1(\lambda)\end{pmatrix}\qquad(S11)$$

And output polarization states can be written as:

$$\widetilde{\mathrm{P}^{-}}=\begin{pmatrix}e^{i\varphi_\alpha(\lambda)} & 0\\ 0 & e^{i\varphi_\beta(\lambda)}\end{pmatrix}\cdot R(-P_2(\lambda)-\theta(\lambda))\cdot\begin{pmatrix}cosP_1(\lambda)\\ i\cdot sinP_1(\lambda)\end{pmatrix}$$

$$=\begin{pmatrix}e^{i\varphi_\alpha(\lambda)} & 0\\ 0 & e^{i\varphi_\beta(\lambda)}\end{pmatrix}\cdot\begin{pmatrix}cos\alpha_\lambda\\ sin\alpha_\lambda\cdot e^{i\beta_\lambda}\end{pmatrix}\qquad(S12)$$

For conjugate polarization conversion, $\widetilde{\mathrm{P}^{-}}=\begin{pmatrix}cos\alpha_\lambda\\ sin\alpha_\lambda\cdot e^{-i\beta_\lambda}\end{pmatrix}$, where $\beta_\lambda=-[\varphi_\beta(\lambda)-\varphi_\alpha(\lambda)]/2$ represents conjugate evolutionary phase. Thus, the relationship between continuous conjugate polarization conversions and the continuous optical responses of meta-atoms can be established:

$$\begin{pmatrix}cos\alpha_\lambda\\ sin\alpha_\lambda\cdot e^{i\beta_\lambda}\end{pmatrix}=R(-P_2(\lambda)-\theta(\lambda))\cdot\begin{pmatrix}cosP_1(\lambda)\\ i\cdot sinP_1(\lambda)\end{pmatrix}$$

$$=\begin{pmatrix}cos(P_2(\lambda)+\theta(\lambda))\,cos\,P_1(\lambda)-i\cdot sin\big(P_2(\lambda)+\theta(\lambda)\big)\,sin\,P_1(\lambda)\\ sin\big(P_2(\lambda)+\theta(\lambda)\big)\,cos\,P_1(\lambda)+i\cdot\cos\,(P_2(\lambda)+\theta(\lambda))\,sin\,P_1(\lambda)\end{pmatrix}\qquad(S13)$$

Thus,

$$\frac{sin\alpha_\lambda}{cos\alpha_\lambda}\cdot e^{i\beta_\lambda}=\frac{sin(P_2(\lambda)+\theta(\lambda))\,cos\,P_1(\lambda)+i\cdot\cos\,(P_2(\lambda)+\theta(\lambda))\,sin\,P_1(\lambda)}{cos(P_2(\lambda)+\theta(\lambda))\,cos\,P_1(\lambda)-i\cdot sin(P_2(\lambda)+\theta(\lambda))\,sin\,P_1(\lambda)}$$

$$=\frac{\sin\big(2P_2(\lambda)+2\theta(\lambda)\big)\cos\big(2P_1(\lambda)\big)+i\cdot\sin\big(2P_1(\lambda)\big)}{1+cos\big(2P_2(\lambda)+2\theta(\lambda)\big)\cos\,(2P_1(\lambda))}\qquad(S14)$$

By equating the real and imaginary parts on both sides, we obtain:

$$tan\left(-\frac{\Delta\varphi(\lambda)}{2}\right)=\frac{tan(2P_1(\lambda))}{sin2(P_2(\lambda)+\theta(\lambda))} \qquad (S15)$$

It relates the metaatoms response to continuous conjugate polarization-wavelength channels conversion. Finally, we can obtain the corresponding conjugate modulation by inversely rotating the output polarization states, $\begin{pmatrix} \cos\theta(\lambda) & \sin\theta(\lambda) \\ -\sin\theta(\lambda) & \cos\theta(\lambda) \end{pmatrix}\cdot \begin{pmatrix} cos\alpha_\lambda \\ sin\alpha_\lambda \cdot e^{-i\beta_\lambda} \end{pmatrix}$.

Note 4. The detailed process of the forward analytical model

According to the flowchart of the continuous information-domain reconstruction framework shown in Fig. 4(a) of the manuscript, the detailed process of constructing the metaatom library is shown in Figure S4. The simulation phase data of X-polarization φ_α and Y-polarization φ_β were obtained by sweeping the length and width of the metaatoms from 0.1 to 1.6 μm with a period of 1.7 μm. Then, φ_α and φ_β were fitted as functions of wavelength, and their birefringent phase retardation ranges are shown in Fig. S4 (a-b). The birefringent retardation $\varphi_\beta - \varphi_\alpha$ representing the continuous dispersion response of the metaatoms (Fig. S4 (c)), is employed to fulfill the birefringent phase retardation requirements for continuous polarization states. These requirements are derived through the forward analytical model, expressed as functions of the polarization ellipticity angle and the rotation angle of the metaatoms, $\tan(\beta_\lambda) = \tan(2P_1(\lambda)) / \sin[2(P_2(\lambda) + \theta(\lambda))]$ (mentioned in the manuscript) shown in Fig. S4 (d).

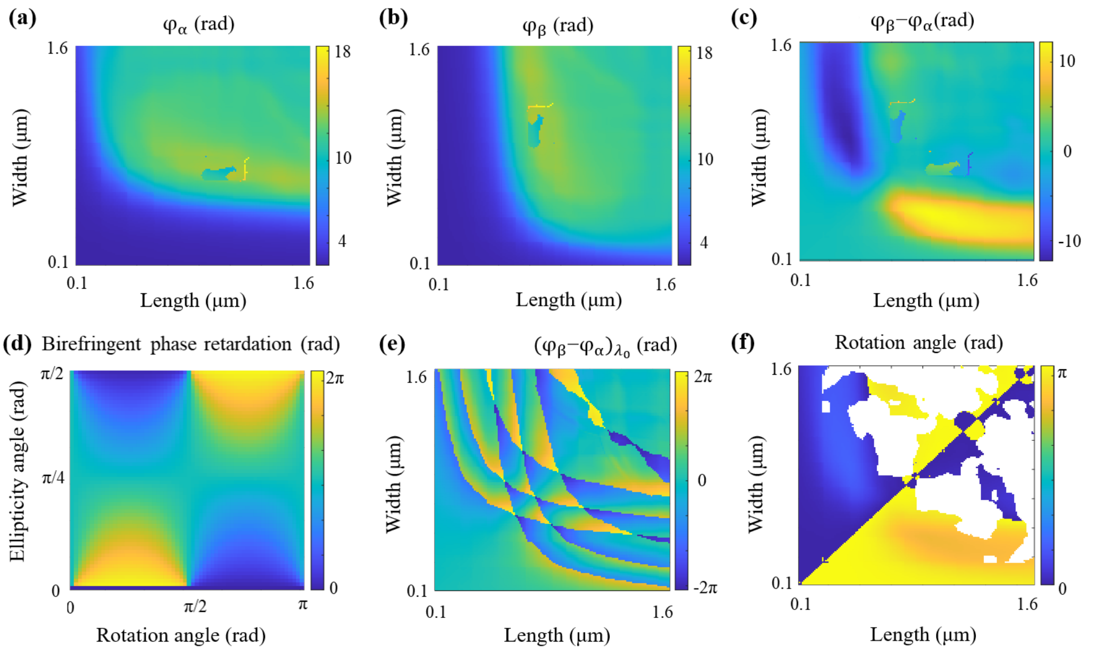

Fig. S4. The building process of the metaatoms library according to the forward analytical model. The dispersion libraries of X-polarization and Y-polarization are represented in (a) and (b), respectively. (c) The birefringent phase retardation range of metaatoms' dispersion response. (d) The required birefringent phase retardation is a function of the continuous ellipticity angle and metaatom's rotation angle. (e) presents the birefringent phase retardation at the central wavelength for all metaatoms in the library. (f) The final metaatoms library with a perturbative factor $\theta(\lambda)$.

Next, it is necessary to optimize the global matching between dispersion and polarization in order to decouple the continuously reconstructed functionalities. During

this process, we evaluate the candidate metaatoms based on their phase and dispersion coverage to identify the optimal matching configuration. The birefringent phase retardation at the central wavelength is employed to tune the global birefringent response, as illustrated in Fig. S4(e). This response serves as an intermediate bridge, enabling alignment between the continuous polarization and dispersion characteristics. Once the matching is finalized, the rotation angles of the metaatoms are employed as a perturbation parameter for further optimization. The initial perturbation is shown in Fig. S4(f). Metaatoms exhibiting transmittance deviations greater than 18% between the X- and Y-polarization channels are excluded. The resulting metaatom library thus consists of elements with perturbative rotation angle and well-matched optical responses.

Note 5. A dimension-interlaced vectorial diffraction neural network for data space compression and nonlocal continuous-domain information reconstruction

As shown in Figure 2(a) in the manuscript, the diffraction neural network consists of an input layer, a hidden layer, and an output layer. The input layer contains the initial complex field distribution, represented as $A_0\exp(i\varphi_0)$, along with continuous inputs corresponding to different wavelengths and polarization states, denoted as P_n. The meta-holography, serving as the hidden layer, is trained to generate multi-channel holographic patterns. The output layer corresponds to the intensity and polarization distribution $A^{'}\exp(i\varphi^{'}P_n^{'})$ on the image plane.

The kernel used in our framework is explicitly based on the Rayleigh–Sommerfeld (R–S) diffraction theory, which accurately describes scalar wave propagation between two arbitrary planes without invoking paraxial or Fresnel approximations. This choice ensures rigorous modeling of broadband and nonparaxial conditions relevant to our mid-infrared metasurface experiments.

The diffraction kernel is expressed as:

$$f(x',y',z,\lambda)=\frac{z}{r^2}\left(\frac{1}{2\pi r}+\frac{1}{i\lambda}\right)\exp\left(\frac{i2\pi r}{\lambda}\right) \tag{S16}$$

where λ denotes the free-space wavelength. z is the propagation distance from the metasurface to the observation plane, and $r=\sqrt{(x'-x)^2+(y'-y)^2+z^2}$ is the diffraction distance between a meta-atom at position (x,y,0) and a sampling point (x', y', z) in the output field.

According to the Huygens–Fresnel principle, every subwavelength element of the metasurface acts as an independent secondary source emitting a spherical wavelet. The global optical field at the observation plane is the coherent superposition of these wavelets, each modulated by the local Jones matrix of the corresponding metaatom. Thus, the output field is expressed as Eq.(4) in the manuscript. This nonparaxial Rayleigh–Sommerfeld formulation was adopted because:

(i) Broadband validity: It remains accurate over a wide spectral range, avoiding the

small-angle limitation of Fresnel or angular-spectrum approximations.

(ii) Subwavelength precision: It preserves near-field phase information, crucial for describing nonlocal coupling between meta-atoms.

(iii) Physical consistency: It enables a one-to-one correspondence between the neural-network "neurons" and physical secondary sources on the metasurface plane, linking the learned diffraction field to real-space wave propagation.

Additionally, since the method is grounded in first-principles diffraction physics rather than data memorization, it generalizes inherently across different wavelengths, polarization bases, and target wavefronts without retraining. In practice, the same framework can be re-used to design metasurfaces for distinct functional targets (e.g., multicolor vectorial holography, continuous achromatic imaging) by simply redefining the objective field distribution.

For conventional metasurface optimization targeting *n(λ)* wavelength channels, each meta-atom must satisfy:

$$\tilde{J}_i\,(\lambda) = R(-\theta_i) \cdot \begin{pmatrix} t_\alpha(\lambda) & 0 \\ 0 & t_\beta(\lambda) \end{pmatrix} \cdot R(\theta_i) \qquad (S17)$$

Where $t_{\alpha/\beta}$ are the complex transmission coefficients of the two birefringent eigen-axes and θ_i is the in-plane rotation. Owing to the lack of a continuously tunable mechanism, traditional methods must independently solve each target polarization–wavelength channel, yielding an overall search space on the order of $4 \times n_\theta \times n_\lambda \times n$ when jointly optimizing across multiple channels and structural candidates. This cubic scaling renders brute-force or grid-based optimization intractable for broadband vectorial control.

In our framework, the wavelength dependence of each eigen-channel transmission is explicitly parameterized rather than solved discretely. The complex transmission functions are approximated by third-order polynomials:

$$t_{\alpha,\beta}(\lambda) = a_0 + a_1\lambda + a_2\lambda^2 + a_3\lambda^3 \qquad (S18)$$

whose real and imaginary parts form eight real coefficients per eigen-axis. The rotation angle θ(λ) with equivalent continuous dispersion is analytically determined from the conjugate-polarization constraint derived in Eq. (S5), leaving these eight coefficients as the only trainable variables. Consequently, the global optimization scales linearly with the library size n, i.e. 8×n, representing a reduction from cubic to linear complexity.

The dimension-interlaced vectorial-diffraction neural network (DVNN) embeds this compressed analytical model within a differentiable pipeline. Each meta-atom is treated as a trainable node whose polynomial coefficients are weights updated by the Adam optimizer to minimize a loss function comparing the simulated far-field $E_{out}(\lambda)$with the target field $E_{target}(\lambda)$:

$$\varepsilon = \sum_{\lambda} \left\| E_{out}(\lambda) - E_{target}(\lambda) \right\|^2 \qquad (S19)$$

After convergence, the optimized coefficients are mapped back to physical geometries through the meta-atom library, uniquely defining the fabrication mask. This formulation transforms the discrete, channel-wise search into a compact continuous-domain optimization that captures both spectral and polarization evolution with greatly reduced computational cost while preserving high accuracy.

Note 6. Analysis of fabrication errors

We analyzed the effects of fabrication tolerances, including sidewall verticality, etch depth, and linewidth deviations, on the design of birefringent dispersion, taking an elliptical nanopillar unit (length: 630 nm; width: 550 nm) as an example, as shown in Fig.S5. (a–d) depict the effects of etch angle on the X-and Y-polarization phases, the birefringent phase delay, and the resulting dispersion range. The inset shows an SEM image of a fabricated unit, confirming the achieved sidewall verticality. As indicated by the dispersion curves in (d), etch angles below 0.5° ensure the dispersion phase shift less than π/4. Considering that our deep silicon etching process can reach an aspect ratio of 23:1, this tolerance requirement is readily achievable. (e–h) illustrate the influence of etch depth. Etch-depth deviations of up to ±200 nm cause only minor phase shifts (~0.1π) without affecting the overall group-delay modulation range. (i–l) show that linewidth variations of ±20 nm induce only slight birefringence shifts (~0.2π). Tolerance analysis shows that deviations in sidewall angle (<0.5°), etch depth (±200 nm), and linewidth (±20 nm) introduce minimal influence on birefringent dispersion and overall holographic performance. These findings confirm that the proposed metasurface design is well within the capability of current nanofabrication technologies.

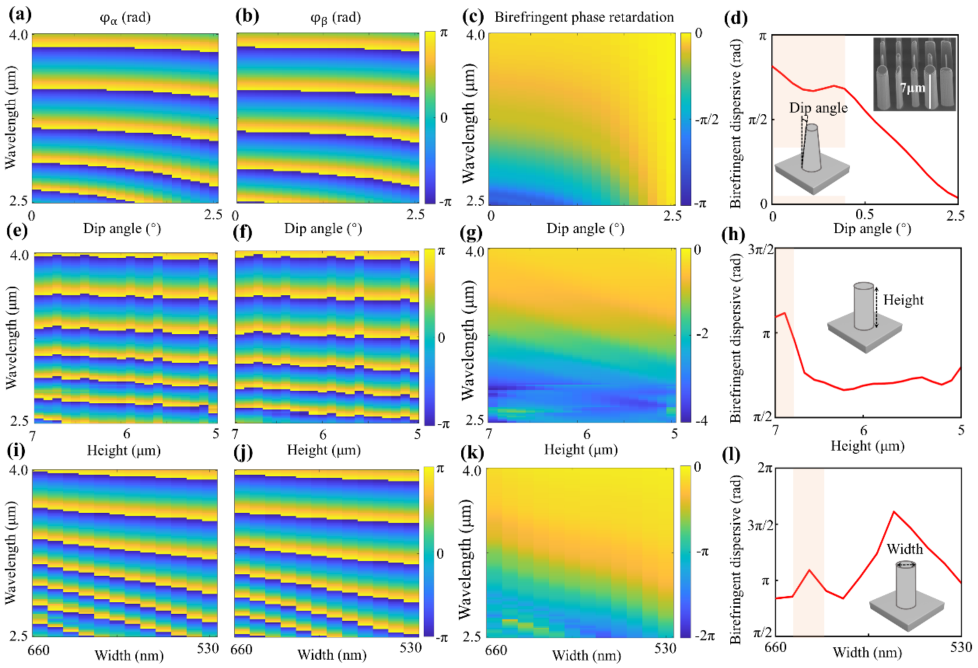

Fig. S5. Tolerance analysis of fabrication errors. (a–d) Effects of etch-angle variation on X-/Y-polarization phase, birefringent phase delay, and dispersion range (inset: SEM showing sidewall verticality). (e–h) Influence of etch-depth deviations.(i–l) Effects of linewidth variation.

Note 7. The fidelity and crosstalk quantitative analysis of metasurface holography

The correlation coefficient is defined as to quantify fidelity:

$$corr(T,R) = \frac{\mathrm{COV}(T,R)}{\sqrt{D(T)\cdot D(R)}} \qquad (S20)$$

where T and R denote the two images, COV(T,R) is their covariance, and D(T) and D(R) are their variances. Fig. S6(a) shows the simulated and experimental correlation coefficients of arbitrary polarization-multiplexed holography. Fig. S6(b) shows the corresponding simulated and experimental results for multicolor vectorial holography. For multicolor vectorial holography, the average experimental correlation coefficient is 0.82 ± 0.01, closely matching the simulated value of 0.94. For arbitrary polarization-multiplexed holography, the mean correlation reaches 0.62 ± 0.08 across five representative wavelengths (2.7, 3.055, 3.35, 3.6 and 3.92 μm). The corresponding comparisons between simulated and experimental correlation maps demonstrate excellent agreement and confirming high-fidelity vectorial reconstruction.

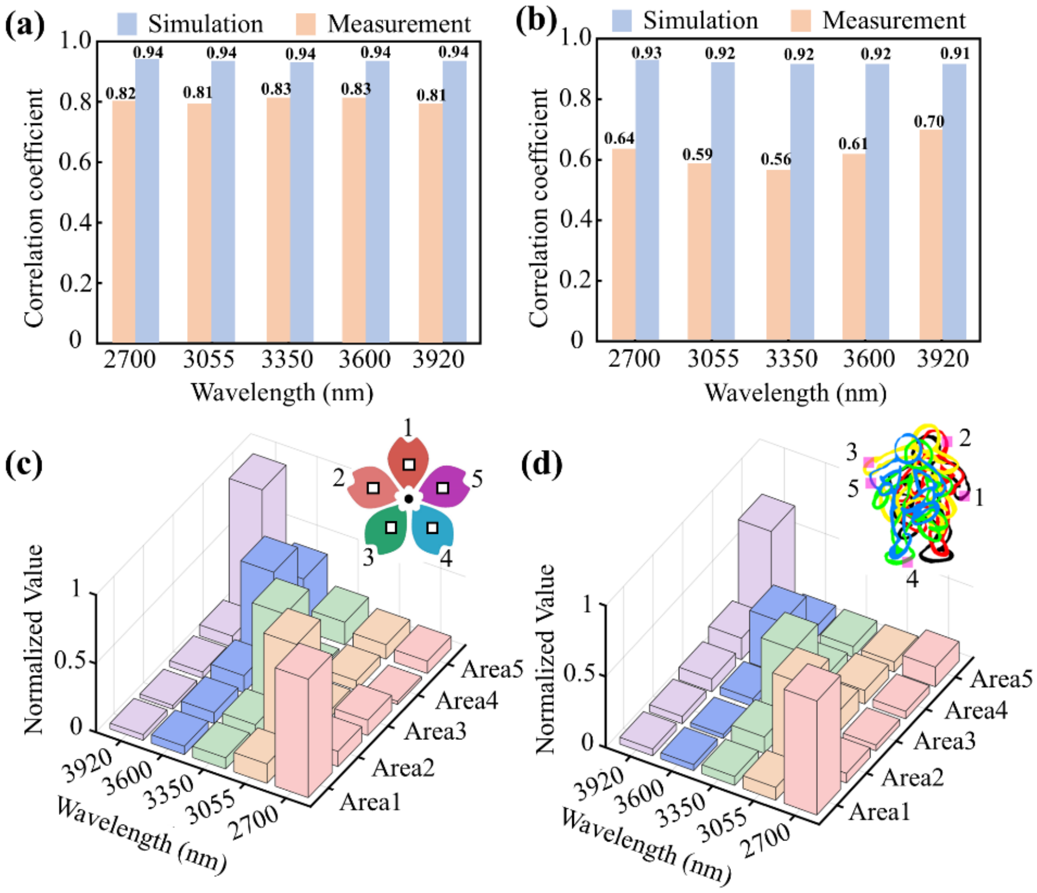

Fig. S6. The fidelity and crosstalk quantitative analysis of arbitrary polarization-multiplexed holography (a, c) and arbitrary polarization-multiplexed holography (b, d). (c-d) are experimental results. Crosstalk is normalized by the maximum energy channel.

To assess crosstalk, five non-overlapping regions were selected, and the energy within each of the five channels was normalized. Fig. S6(c) displays the experimentally measured crosstalk for arbitrary polarization-multiplexed holography, and Fig. S6(d) illustrates the crosstalk results for multicolor vectorial holography. The average inter-channel crosstalk is below 0.16, with the maximum value below 0.29, indicating strong channel isolation. These results collectively confirm the high-fidelity reconstruction and low inter channel crosstalk performance achieved by our device.

Note 8. Expansion of non-degenerate multicolor holography

Here, we present simulation results of multi-channal multiplexed achromatic holograms with ten independent channel combinations, as shown in Fig. S7. In these designs, the input and output polarization channels are conjugate and vary continuously with the wavelength. It is important to note that achieving ten independent holographic images across ten distinct wavelengths and polarization channels does not represent the upper limit of our approach. To ensure sufficient channel isolation, we demonstrate only ten multiplexed holograms in this work. We emphasize that the information capacity of metasurface holography can be further extended by increasing the structure height, optimizing the modal topology of the meta-atoms, or enlarging the metasurface area.

In fact, the information capacity of the metasurface is fundamentally bounded by the maximum achievable group delay span ($\Delta\tau$), which dictates the total phase accumulation range available for spectral encoding. Increasing the pillar height allows multiple guided modes to coexist within each unit cell, effectively enhancing the modal density $N_m \propto \mathrm{H}/\lambda_{eff}$ and thus expanding the available $\Delta\tau$. This enables finer phase dispersion control and supports more wavelength channels before modal overlap induces crosstalk. However, this improvement comes at the cost of more demanding fabrication. High-aspect-ratio pillars (typically >20:1) require deep etching and precise sidewall control to prevent collapse or tapering, especially for large-area arrays. In the mid-infrared, silicon can achieve aspect ratios up to ~30:1 using cryogenic DRIE processes, while visible or near-infrared implementations in GaN (~17:1) and TiO_2 (~37:1) remain challenging. Such constraints limit the practically achievable number of fully independent channels and impose upper bounds on the design parameter space. Additionally, In broadband operation, absorption and scattering losses accumulate across longer optical paths within tall structures. This limits the attainable Q-factor of guided modes and may distort the wavelength-dependent polarization response. For example, in Si at 3–5 μm, absorption losses are <0.2 cm^{-1} but increase significantly toward shorter wavelengths, reducing efficiency for near-infrared operation. Therefore, optimization must balance spectral bandwidth with acceptable insertion loss, depending on the material platform.

Additionally, in conventional birefringent meta-atoms, the Jones matrix eigenstates

are constrained to the equatorial plane of the Poincaré sphere, restricting polarization encoding to a single geometric degree of freedom (the rotation angle). By engineering multi-resonant or topologically nontrivial geometries, one can lift this constraint and access a higher-dimensional eigen-polarization manifold where the eigenvectors span the full sphere. This expands the available polarization state space and allows independent modulation of orthogonal spin channels or elliptical states, thereby increasing the number of usable polarization–wavelength combinations. However, as the geometric parameter space grows, optimization convergence slows and tolerance sensitivity increases. On the other hand, composite or freeform meta-atoms introduce exponential growth in structural parameters, drastically expanding the optimization database and increasing sensitivity to fabrication errors. As geometry complexity rises, ensuring consistent performance under ±10 nm dimensional deviations becomes increasingly difficult.

Finally, the number of distinct holographic channels is also proportional to the Fourier-space support of the aperture. A larger metasurface provides a finer sampling of spatial frequencies and a higher diffraction-limited resolution, enabling independent manipulation of more encoded images or spectral–polarization channels. Quantitatively, the spatial bandwidth $B \propto D/\lambda z$ (where D is the aperture diameter and z the propagation distance) determines the number of orthogonal far-field modes that can be formed. Nevertheless, increasing D also raises fabrication cost and alignment complexity, so the device size should be optimized to balance channel capacity and practical constraints.

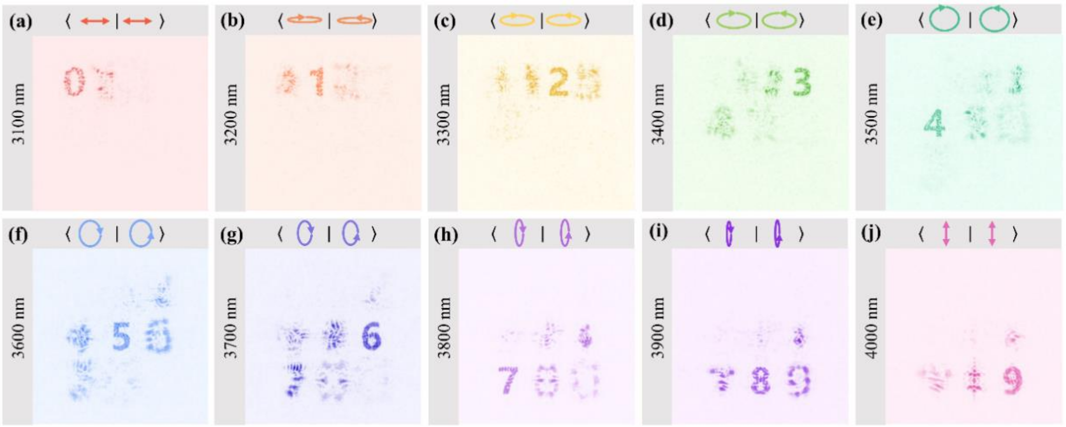

Fig. S7. The simulation of multi-channel holography with 10 densely sampled continuous polarization channels at 10 wavelengths. The wavelengths are sampled in the range of 3.1–4 μm with a smaller interval of 100 nm, while the polarization states are continuously and densely sampled from X- to Y-polarization at 10° intervals of polarization angle.

Note 9. Expansion of non-degenerate multicolor holography

To further validate the "continuity" mentioned by the theory, we conducted both interpolated simulations and experiments on the continuous-gradient achromatic hologram shown in Figure 5. Simulations were performed for input–output channels at 1° angular intervals and 15 nm wavelength steps. The measured output polarizations vary smoothly with wavelength, represented by red stars on the Poincaré sphere, while the black circles indicate the theoretical predictions, as shown in Fig. S8(a). Fig. S8(b) shows the simulated polarization angles under different channels, in agreement with the target values indicated by the black dashed lines. The high resolution in controlling polarization dispersion provides clear evidence for the "continuity" described. Moreover, we conducted three interpolation experiments by introducing additional polarization and wavelength channels using polarizer and band-pass filters, as highlighted in the red box in the Fig.S9. These experimental results substantiate the feasibility of continuous channel interpolation. We believe that the continuous wavelength-dependent tunability of the polarization channels could be further validated with a sufficient number of filtering elements.

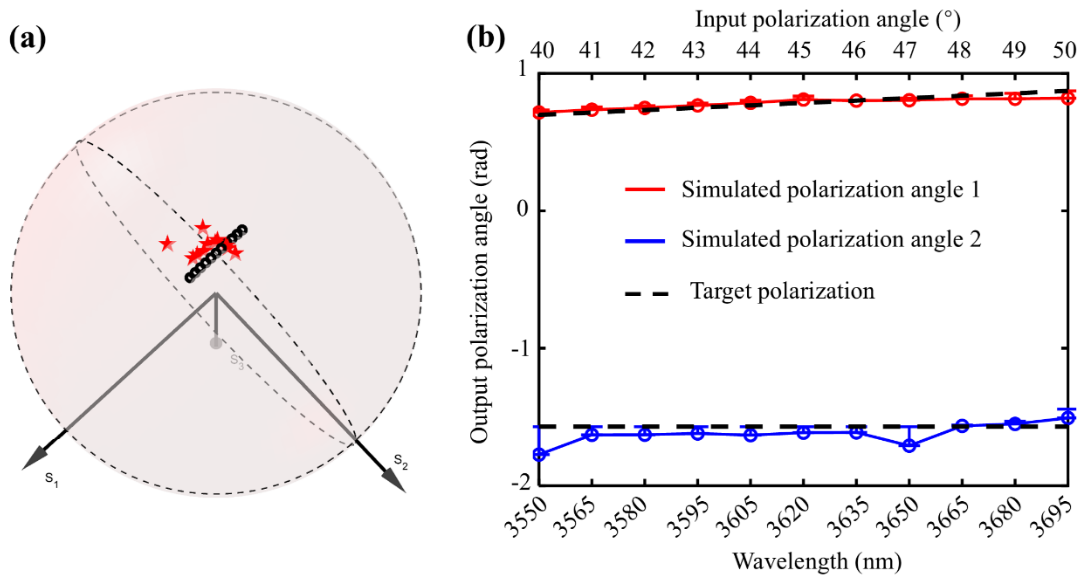

Fig. S8. The interpolated simulation of the continuous-gradient achromatic hologram. (a) The target (black circle) and simulated (red star) output Stokes parameters on the Poincaré sphere. (b) The target (black dashed lines) and simulated polarization angles (red and blue lines) under different channels. The system was sampled at 1° polarization and 15 nm wavelength intervals.

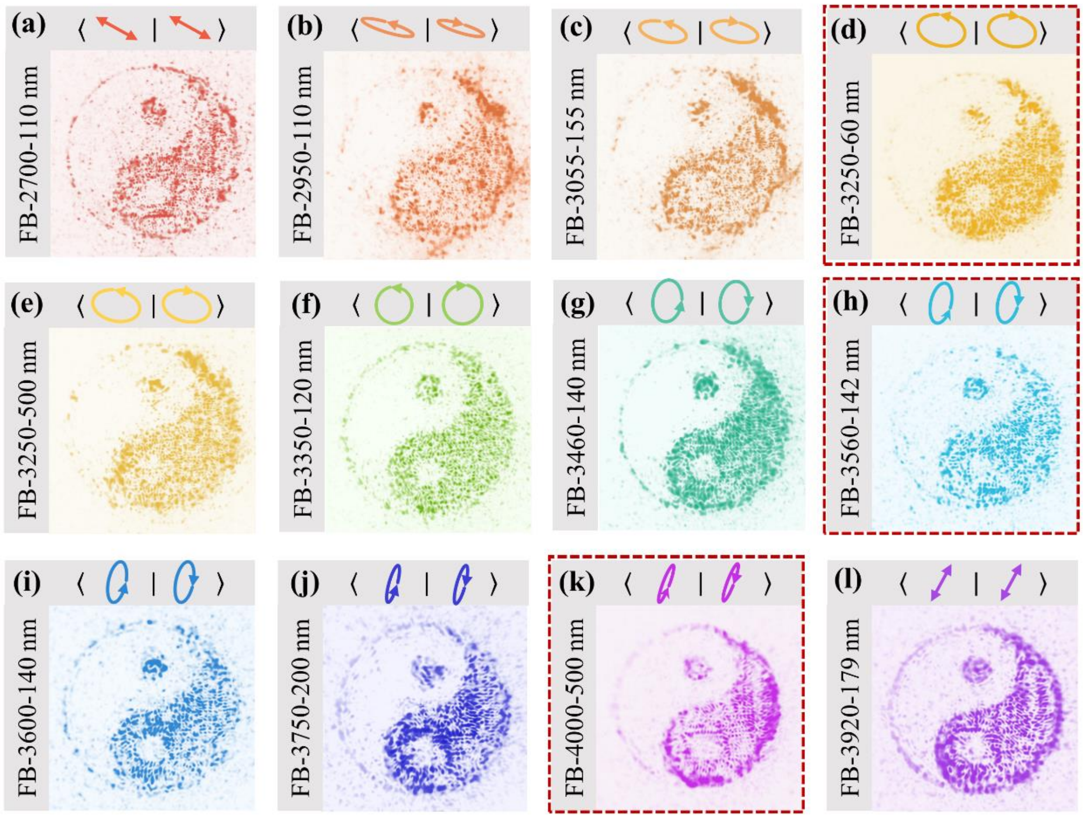

Fig. S9. The experimental characterization of broadband continuous-gradient polarization holography. Channel interpolation was verified using mid-infrared bandpass filters under continuously varying input and output polarization channels, as highlighted in the red box. The wavelengths are densely sampled within the 2.7–4 μm range according to the optical filters available to us. The conjugate-polarized input–output pairs are mapped on the Poincare sphere as shown in Fig.5(a)

Note 10. Quantitative comparisons between different design methods

We conducted a comparative study of three representative holographic multiplexing strategies—(i) spatially interleaved metasurfaces, (ii) multifocal-plane designs, and (iii) our proposed nonlocal metasurface framework—using the same target images and material parameters. The results shown in Fig. S10(a) reveal that our nonlocal design achieves fidelity levels comparable to or exceeding those of the multifocal-plane method (corr ≈ 0.80–0.86) while eliminating the need for multiple observation planes. The interleaved approach shows reduced fidelity (corr ≈ 0.59) due to spatial sampling gaps and near-field interference between unit-cell groups. As shown in Fig. S10(b), the relative energy efficiency of our device is higher than that of interleaved metasurfaces and comparable to that of multifocal-plane designs. The improvement arises from the suppression of redundant diffraction channels and optimized nonlocal redistribution of field energy. We evaluated inter-channel crosstalk by integrating the normalized energy leakage between five independent image regions [Fig. S10(c–f)]. The average crosstalk level of our metasurface remains below 16%, approximately 12% lower than the interleaved benchmark. These results confirm that the proposed method achieves high fidelity, high efficiency, and strong channel isolation in a single planar configuration.

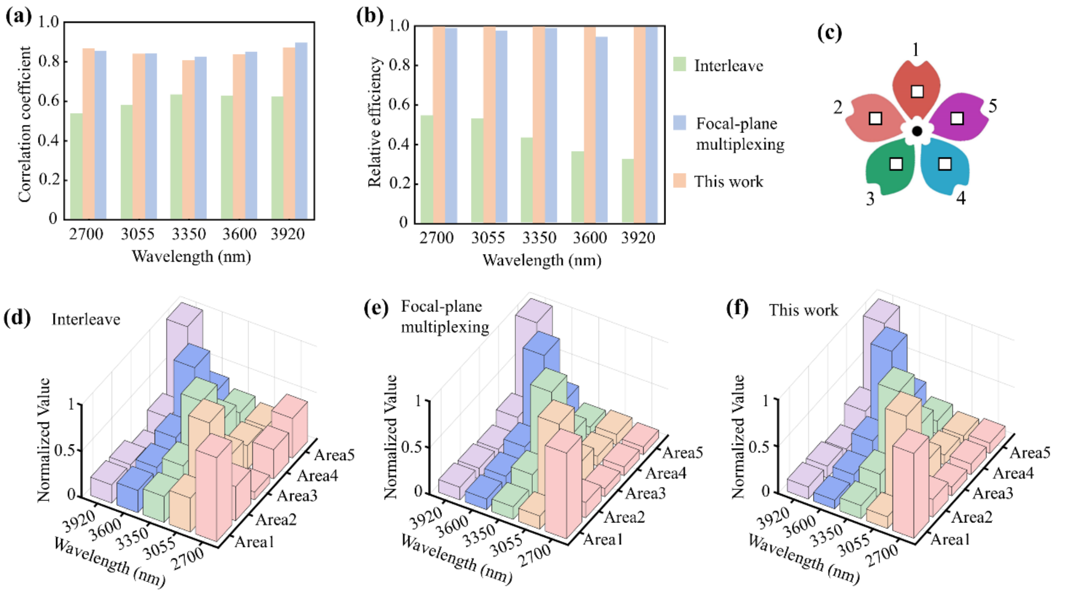

Fig. S10. The fidelity (a), efficiency (b), and crosstalk (d-f) quantitative analysis of three holographic multiplexing strategies. The relative energy efficiency is normalized by three strategies. Crosstalk is normalized between different channels within each region. (c) is the five independent image regions for energy normalization.
